# PDK1 regulates the survival of the developing cortical interneurons

**DOI:** 10.1186/s13041-020-00604-6

**Published:** 2020-05-04

**Authors:** Yongjie Wei, Xiaoning Han, Chunjie Zhao

**Affiliations:** Key Laboratory of Developmental Genes and Human Diseases, MOE, School of Medicine, Southeast University, Nanjing, 210009 China

**Keywords:** Interneuron, Apoptosis, Cell migration, Cell proliferation, PDK1, AKT signal, Telencephalon

## Abstract

Inhibitory interneurons are critical for maintaining the excitatory/inhibitory balance. During the development cortical interneurons originate from the ganglionic eminence and arrive at the dorsal cortex through two tangential migration routes. However, the mechanisms underlying the development of cortical interneurons remain unclear. 3-Phosphoinositide-dependent protein kinase-1 (PDK1) has been shown to be involved in a variety of biological processes, including cell proliferation and migration, and plays an important role in the neurogenesis of cortical excitatory neurons. However, the function of PDK1 in interneurons is still unclear. Here, we reported that the disruption of *Pdk1* in the subpallium achieved by crossing the *Dlx5/6-Cre-IRES-EGFP* line with *Pdk1*^*fl/fl*^ mice led to the severely increased apoptosis of immature interneurons, subsequently resulting in a remarkable reduction in cortical interneurons. However, the tangential migration, progenitor pools and cell proliferation were not affected by the disruption of *Pdk1*. We further found the activity of AKT-GSK3β signaling pathway was decreased after *Pdk1* deletion, suggesting it might be involved in the regulation of the survival of cortical interneurons. These results provide new insights into the function of PDK1 in the development of the telencephalon.

## Introduction

The cognitive function of the cerebral cortex relies on highly organized neural circuits that consist of excitatory neurons and γ-aminobutyric acid (GABA) ergic inhibitory interneurons. Despite comprising only 20–30% of the cortical neurons, interneurons play crucial roles in maintaining the balance between excitation and inhibition (E/I) and modulating cortical function [1–5]. Previous studies have demonstrated that cortical interneuron abnormalities are closely associated with a variety of neuropsychiatric disorders, such as schizophrenia, epilepsy, and autism spectrum disorders [6–9]. During the development cortical interneurons originate from the ganglionic eminence in the ventral telencephalon [10–12]. Somatostatin (SST)- and parvalbumin (PV)-expressing interneurons are derived from the medial ganglionic eminence (MGE) [11], while the caudal ganglionic eminence (CGE) produces PROX1-expressing interneurons including vasoactive intestinal peptide (VIP^+^) and Reelin^+^ interneurons [13, 14]. Newborn interneurons migrate to the dorsal cortex following two well-defined tangential routes: a superficial route in the marginal zone (MZ) and another route in the intermediate zone/subventricular zones (SVZ) [15, 16]. After reaching the dorsal cortex, interneurons subsequently undergo radial migration to populate to the final destinations and establish connections with their targets, forming functional neural circuits [17, 18]. However, the mechanisms underlying the development of cortical interneurons are still poorly understood.

The serine/threonine kinase 3-Phosphoinositide-dependent protein kinase-1 (PDK1) belongs to the cAMP-dependent, cGMP-dependent and protein kinase C (AGC) kinase family [19–22]. PDK1, the key downstream effector of PI3K signaling that is activated by various extracellular stimuli, is involved in many biological processes, such as cell proliferation and growth, neural differentiation, cell migration, cell polarization and cell survival [23–28]. Conditional deletion of *Pdk1* results in increased apoptosis and migration defects in excitatory neurons [29, 30]. Previously, we reported that PDK1 controls the transition of apical progenitors to basal progenitors through regulating asymmetric cell division, causing subsequent increased cell proliferation and neural output in the developing cortex [31]. We also found that the loss of PDK1 in the developing dentate gyrus causes unbalanced cell proliferation and neurogenesis [32]. A growing amount of evidence suggests that PDK1 is upregulated in cancer cells and required for cell survival [33–35]. All these studies demonstrate that PDK1 plays different roles during different biological processes. Previously, Oishi K et al. reported that PDK1 promotes the differentiation of neuronal progenitor cells (NPCs) into GABAergic interneurons but not glutamatergic neurons [36]. However, the precise role of PDK1 in cortical interneuron development is not well understood. In this study, we found that the conditional deletion of *Pdk1* in the subpallium resulted in the severely increased apoptosis of immature interneurons and subsequently led to a significantly decreased number of cortical interneurons. No obvious defects in tangential migration and cell proliferation were detected. We further demonstrated that PDK1 regulates the survival of the developing cortical interneurons.

## Materials and methods

### Animals

The *Pdk1*^*fl/fl*^ mice [37] were obtained from Dr. Zhongzhou Yang (Model Animal Research Center of Nanjing University) and Dr. Jun Gao (Nanjing Medical University at Nanjing). Conditional disruption of *Pdk1* in the subpallium was achieved by crossing the *Dlx5/6-Cre-IRES-EGFP* line [38] with *Pdk1*^*fl/fl*^ mice. The *Dlx5/6-Cre-IRES-EGFP; Pdk1*^*fl/fl*^ mice are referred to as the *Pdk1* cKO mice. The *Pdk1*^*fl/fl*^ and *Dlx5/6-Cre-IRES-EGFP; Pdk1*^*fl/+*^ mice are referred to as control mice. The primer pairs 5′- ATCCCAAGTTAC TG AGTTGTGTTGGAA G-3′ and 5′- TGTGGACAAACAGCAATGAACAT ACACGC-3′, which amplified a 202-bp band for the control and a 236-bp band for *Pdk1* cKO were used. All mice were maintained on a C57/B6 background and bred in the animal facility of Southeast University (Nanjing, Jiangsu, China). The day of vaginal plug detection was defined as embryonic day (E) 0.5, and the day of birth was defined as postnatal day 0 (P0). All experiments were performed according to the guidelines approved by Southeast University.

### Quantitative real-time PCR

Total RNA from the GE at E16.5 was isolated using an RNeasy Plus mini kit for RNA isolation (Qiagen, Cat. No. 74134) according to the manufacturer’s instructions [39]. Each sample was reverse transcribed using a PrimeScript™ RT reagent kit (TakaRa, Cat. No. RR047A). Quantitative real-time PCR reactions was performed using SYBR Green fluorescent master mix (Roche, Cat. No. 04707516001) on a StepOne-Plus Real-Time PCR System (Applied Biosystems). Relative gene expression was compared between samples after the expression was normalized to the expression of the most reliable endogenous gene (glyceraldehyde 3-phosphate dehydrogenase, *Gapdh*). At least three brain pairs were obtained from 3 different litters and used for analysis. Statistical analysis was performed using Student’s t-test. The primers used are listed in Table 1.

**Table 1:** Primers used for real-time PCR

Gene	Forward	Reverse	Tm(°C)
*Gapdh*	ACCACAGTCCATGCCATCAT	GTCCACCACCCTGTTGCTGTA	62
*Pdk1*	AACTGGCCACTTCCAGAGAA	AAAGAAGGGGTGATCCAGGC	62

### Western blotting

GEs were collected at E16.5 and prepared as described previously [32]. Statistical analysis was performed using Student’s t-test. The PDK1 antibody is listed in Table 2.

**Table 2:** Antibodies used for immunostaining

Antibody	Host species	Source	Catalog number	Dilutions
PDK1	Rabbit	Epitomic	3377–1	1:1000
GAPDH	Rabbit	CST	5174S	1:5000
AKT	Rabbit	CST	9272S	1:1000
p-AKT^Thr308^	Rabbit	CST	13038P	1:500
p-AKT^Ser473^	Rabbit	CST	4060S	1:1000
GSK3β	Rabbit	CST	9315S	1:2000
p-GSK3β^Ser9^	Rabbit	CST	5558S	1:1000
PTEN	Rabbit	CST	9188S	1:1000
PROX1	Goat	R&D	AF2727	1:250
Ascl1	Rabbit	Santa Cruz	sc-28,688	1:250
Olig2	Rabbit	Millipore	AB9610	1:500
PH3	Rat	Abcam	ab10543	1:500
BrdU	Rat	Abcam	ab6326	1:1000
Ki67	Rabbit	Abcam	ab16667	1:100
Cleaved caspase-3	Rabbit	Millipore	AB3623	1:1000
HRP-linked anti-rabbit IgG		CST	7074	1:5000
Alexa Fluor 488 goat anti-chicken		Molecular Probes	A11039	1:500
Alexa Fluor 488 donkey anti-rabbit		Molecular Probes	A11008	1:500
Alexa Fluor 633 goat anti-rabbit		Molecular Probes	A21071	1:500
Alexa Fluor 546 donkey anti-rabbit		Molecular Probes	A10040	1:500
Alexa Fluor 488 goat anti-rat		Molecular Probes	A11006	1:500
Alexa Fluor 546 goat anti-rat		Molecular Probes	A11081	1:500
Alexa Fluor 633 goat anti-rat		Molecular Probes	A21094	1:500
Alexa Fluor 546 rabbit anti-goat		Molecular Probes	A21085	1:500

### Immunostaining

Brains were fixed by transcardiac perfusion with PBS followed by cold 4% paraformaldehyde (PFA). The brains were then postfixed overnight, cryoprotected in 30% sucrose at 4 °C for 12 to 16 h, and then embedded in optimum cutting temperature (OCT) compound. Brains at embryonic stages were coronally sectioned to 12 μm, while those at postnatal stages were coronally sectioned to 25 μm using a Leica CM 1950 cryostat. Immunohistochemical analysis was then performed as previously reported [40]. The antibodies used are listed in Table 2. DAPI was purchased from Sigma-Aldrich (D9564).

### In situ hybridization

Brains were harvested, fixed and hybridized as previously described [41, 42]. Information regarding the primers used to generate probes for *Sst*, *Pv*, and *CyclinD2* are listed in Table 3.

**Table 3:** Primers used to synthesize the probes used for in situ hybridization

Gene	Forward	Reverse	L (bp)
*Sst*	CCGGAATTCACGCTACCGAAGCCGTC	ACGCGTCGACGGGGCCAGGAGTTAAGGA	500
*Pv*	GGACATCAAGAAGGCGATAGG	TCATCCGAGGGCCATAGAG	450
*CycinD2*	CCGGAATTCGGACCGTTTCTTGGCTGGAG	ACGCGTCGACGCTCTGTCAGGGCATCACAC	440

### BrdU administration

5-Bromo-2-deoxyUridine (BrdU, Sigma-Aldrich) was dissolved in physiological saline at a concentration of 10 mg/ml. A single intraperitoneal injection of BrdU (50 mg/kg) was administered to pregnant mice at E12.5, E14.5, and E16.5. Then, embryonic brains were then harvested 0.5 h after the injection for acute labeling analysis.

### Microscopy and image analysis

The tissue sections were viewed under a confocal microscope (Olympus FV1000), and images were collected and analyzed with FV10-ASW image analysis software.

### Statistical analysis

**Fig. 1:**
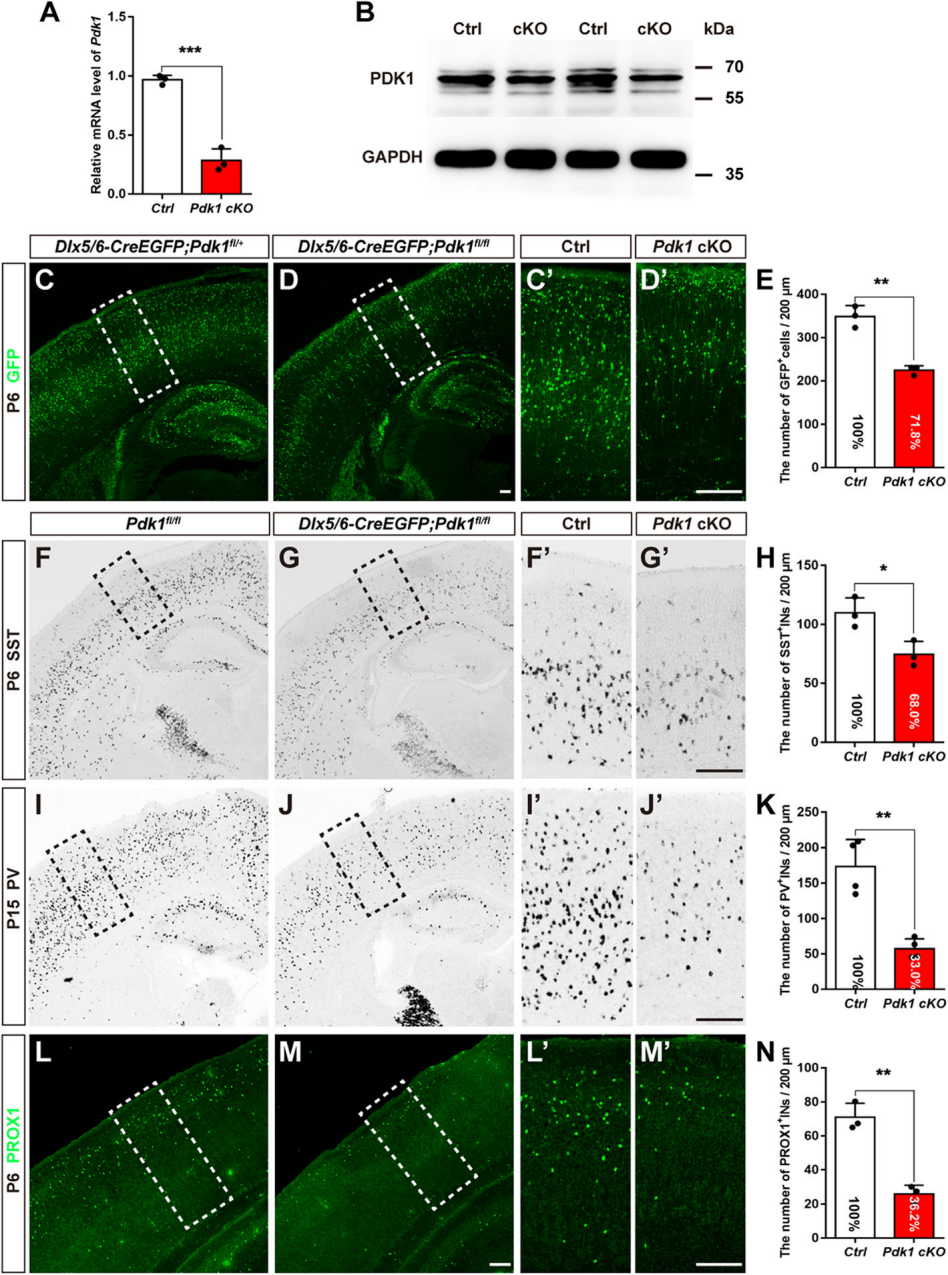
The reduction in cortical interneurons in the postnatal cortex after *Pdk1* ablation. (**a**) Real-time PCR showed that the level of *Pdk1* transcription was decreased in the *Pdk1* cKO subpallium at E16.5 (Ctrl: *N* = 4, *Pdk1* cKO: N = 4, *P* = 0.004, Student’s t-test, with alpha = 0.05). *Gapdh* was used as an internal control. (**b**) Western blot analysis revealed decreased levels of PDK1 in the *Pdk1* cKO subpallium at E16.5 (N = 4). GAPDH was used as an internal control. (**c-d***’*) Immunostaining for GFP showed that the total number of cortical interneurons in the *Pdk1* cKO cortex was decreased at P6. (**e**) Statistical analysis of GFP^+^ interneurons (Ctrl: *N* = 3, *Pdk1* cKO: N = 3, *P* = 0.0013, Student’s t-test, with alpha = 0.05). (**f-g’**) In situ hybridization of SST showed a reduction in SST^+^ interneurons in the *Pdk1* cKO cortex at P6. (**h**) Statistical analysis of SST^+^ interneurons (Ctrl: N = 3, *Pdk1* cKO: N = 3, *P* = 0.023, Student’s t-test, with alpha = 0.05). (**i-j’**) In situ hybridization of PV at P15. (**k**) Statistical analysis of PV^+^ interneurons (Ctrl: N = 4, *Pdk1* cKO: N = 4, *P* = 0.0013, Student’s t-test, with alpha = 0.05). (**l-m’**) Immunostaining for PROX1 revealed that the number of CGE-derived cortical interneurons in the *Pdk1* cKO cortex was decreased at P6. (**n**) Statistical analysis of PROX1^+^ interneurons (Ctrl: N = 3, *Pdk1* cKO: N = 3, *P* = 0.0013, Student’s t-test, with alpha = 0.05). The data are presented as the mean ± SEM. Scale bar, 100 μm

**Fig. 2:**
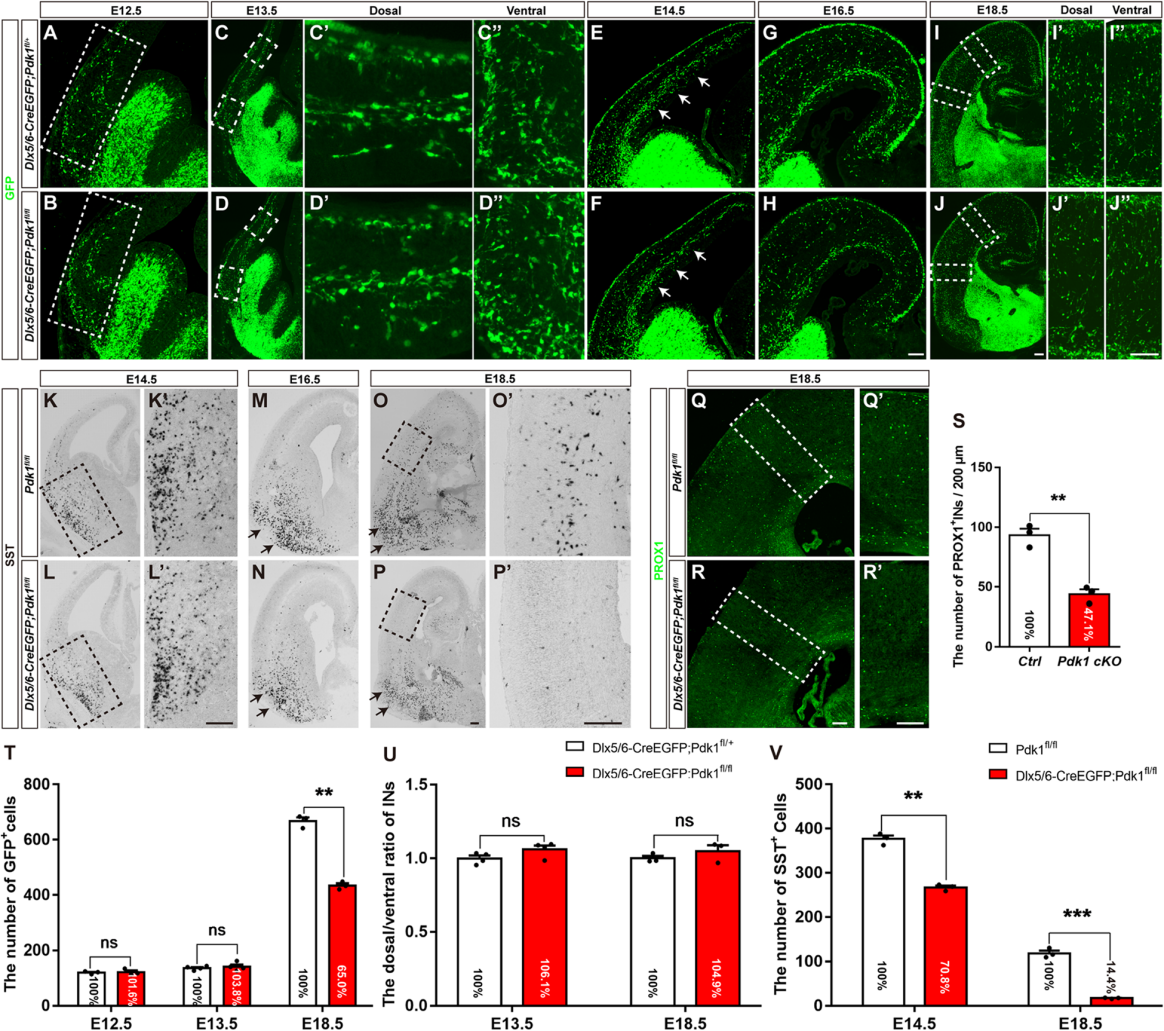
Embryonic loss of cortical interneurons with unchanged tangential migration after *Pdk1* deletion. (**a-d***”*) Immunofluorescence for GFP in coronal sections showed comparable distribution patterns and numbers of cortical interneurons at E12.5 and E13.5 between *Pdk1* cKO and control mice. (**e-f**) Immunofluorescence for GFP revealed that the number of GFP^+^ cortical interneurons in the SVZ was reduced in the *Pdk1* cKO cortex at E14.5. (**g-j***”*) The number of GFP^+^ cortical interneurons in the *Pdk1* cKO cortex gradually decreased from E16.5 to E18.5. (**t**) Statistical analysis of GFP^+^ interneurons (E12.5 Ctrl: N = 3, *Pdk1* cKO: N = 3, *P* = 0.9912; E13.5 Ctrl: N = 4, *Pdk1* cKO: N = 4, *P* = 0.9089; E18.5 Ctrl: N = 3, *Pdk1* cKO: N = 3, *P* = 0.00034, Multiple t-test, with alpha = 0.05/3 = 0.0167). (**u**) Statistical analysis the dorsal/ventral ratio of the numbers of GFP^+^ interneurons between *Pdk1* cKO and control mice at E13.5 and E18.5 (E13.5 Ctrl: N = 4, *Pdk1* cKO: N = 4, *P* = 0.1945; E18.5 Ctrl: N = 3, *Pdk1* cKO: N = 3, *P* = 0.5371, Multiple t-test, with alpha = 0.05/2 = 0.025). (**k-n**) In situ hybridization showed that the number of SST^+^ interneurons in the *Pdk1* cKO subpallium gradually decreased from E14.5 to E16.5. (**o-p***′*) The number of SST^+^ interneurons in the *Pdk1* cKO cortex was decreased at E18.5. (*V*) Statistical analysis of SST^+^ interneurons (E14.5 Ctrl: N = 3, *Pdk1* cKO: N = 3, *P* = 0.00045; E18.5 Ctrl: N = 3, *Pdk1* cKO: N = 3, *P* = 0.00016, Multiple t-test, with alpha = 0.05/2 = 0.025). (**q-r***’*) Immunofluorescence for PROX1 showed that the number of PROX1^+^ cortical interneurons was decreased in the *Pdk1* cKO cortex at E18.5. (**s**) Statistical analysis of PROX1^+^ interneurons (Ctrl: N = 3; *Pdk1* cKO: N = 3; *P* = 0.0018, Student’s t-test, with alpha = 0.05). The data are presented as the mean ± SEM. Scale bar, 100 μm

Student’s *t*-tests were performed to compare changes in the developing telencephlon in absolute and relative percentages of cell numbers. To measure GFP^+^, SST^+^, PV^+^, and PROX1^+^ cells at P6 and P15, and PROX1^+^ cells at E18.5 brains, a minimum of 3 successive coronal sections were examined at the level at which the hippocampal structure is almost horizontal. Cells within a radial column of 200 μm width and spanning from the pial surface to the white matter in the somatosensory cortex were counted. For the measurement of GFP^+^ cells, cells in the whole E12.5 dorsal cortex, a 150 μm × 200 μm area in the dorsal cortex at E13.5 and a 150 μm × 600 μm area at E18.5 were counted. For SST^+^ cells, the whole ventral telencephalon at E14.5 and an area with 800 μm × 600 μm in the dorsal cortex at E18.5 were examined, respectively. For tangential migration ability analysis, a minimum of 3 successive coronal sections at the level at which the hippocampal structure is almost horizontal were selected at E18.5, GFP^+^ cells were counted in a 150 μm × 600 μm area near the pallia-subpallium boundery in the ventral telencephalon and an 150 μm × 600 μm area in the dorsal cortex were examined. Similar dorsal/ventral ratio analysis was performed at E13.5 with a 150 μm × 200 μm area examined. To measure cell proliferation, PH3^+^, BrdU^+^, and Ki67^+^ cells in the MGE were counted at E12.5 (in a 100 μm × 500 μm area), E14.5150 μm × 600 μm), and E16.5 (150 μm × 320 μm), respectively. As for cell apoptosis, at P6 a minimum of 3 successive coronal sections at the level at which the hippocampal structure is almost horizontal were selected, caspase3^+^ cells were then counted in the whole dorsal cortex. At embryonic stages of E12.5, E14.5 and E16.5, caspase3^+^ cells were counted in the whole subpallium. The caspase3^+^ cells in controls were normalized to 100%. All quantitative results are shown as the mean ± standard error of the mean (SEM). Student’s t-tests were used to analyze the level of *Pdk1* transcription at E16.5 (Fig. 1a), the total number of GFP^+^ cortical interneurons at P6 (Fig. 1e), the number of SST^+^ interneurons at P6 (Fig. 1h), the number of PV^+^ interneurons at P15 (Fig. 1k), the number of PROX1^+^ interneurons at P6 and E18.5 (Fig. 1 and 2n and s), and the relative protein expression levels of AKT-GSK3β pathway (Fig. 6b、6D、6F、6H 、6 J and 6 L). Significance for comparisons was defined by Student’s t-tests as follows: *P < 0.05, **P < 0.01, and ***P < 0.001. Values of *P* < 0.05 indicated statistically significant differences. Multiple t-tests were used to analyze the number of GFP^+^ interneurons at E12.5, E13.5 and E18.5 (Fig. 2t), the dorsal/ventral ratio of the numbers of GFP^+^ interneurons at E13.5 and E18.5 (Fig. 2u), the number of SST^+^ interneurons at E14.5 and E18.5 (Fig. 2v), the number of PH3^+^, BrdU^+^ and Ki67^+^ cells at E12.5, E14.5 and E16.5 (Fig. 4s、4T and 4 U), and the number of Caspase-3^+^ cells at E12.5, E14.5, E16.5 and P6 (Fig. 5i). Bonferroni correction was applied to multiple t-tests in this study. Significance for comparisons was defined by Multiple t-tests as follows: *P < 0.0332, **P < 0.0021, ***P < 0.0002, and ****P < 0.0001. Values of *P* < 0.05/n (n = t-tests were done times) indicated statistically significant differences. At least 3 paired brains from 3 different litters were used for each analysis.

## Results

### Loss of *Pdk1* results in a severe reduction in cortical interneurons at postnatal stages

As a number of AGC protein kinase family members, PDK1 has been reported to be ubiquitously expressed [26]. To investigate its role in the development of cortical interneurons, the *Pdk1*^*fl/fl*^ line was crossed with *Dlx5/6-Cre-IRES-EGFP* mice [38] to conditionally delete *Pdk1* in the subpallium. Quantitative real-time PCR showed an approximate 80% deletion efficiency in the GE (Fig. 1a). As for western blot, the PDK1 antibody detected total three including one stronger and two weaker bands between the range of 55kD and 70kD. The stronger band showed a significant reduction in the *Pdk1* cKO subpallium compared to control (Fig. 1b). We have tested several other commercial PDK1 antibodies but found they do not work well especially at developmental stages with nonspecific staining in *Pdk1* cKO tissues. Here considering the *Dlx5/6-Cre* is a powerful tool to delete genes in SVZ progenitors in the ventral telencephalon [14], and the *Pdk1*^*fl/fl*^ line has been employed to disrupt *Pdk1* in several studies [30–32], combined with the result of Quantitative real-time PCR, *Pdk1* could be considered to be efficiently deleted. Most *Pdk1* cKO mice died shortly after birth, with a very small number surviving to P15. We next quantified the total number of GFP^+^ cells, which represented the cortical interneurons, in the postnatal somatosensory cortex. As shown in Fig. 1c-d’, a large number of GFP^+^ interneurons populated to the cortex in control mice, while the number was significantly decreased in the *Pdk1* cKO cortex. Statistical analysis showed an approximately 28.2% reduction in GFP^+^ interneurons in the *Pdk1* cKO cortex compared to the control cortex (Fig. 1e).

To further examine the changes in distinct subgroups of cortical interneurons, we performed in situ hybridization to detect Somatostatin (SST)- and parvalbumin (PV)-expressing cells, which represented MGE-derived interneurons, and immunostaining for PROX1, a specific marker of CGE-derived interneurons. At P6, many SST^+^ interneurons were observed in the control cortex, which exhibited a superficial layer^less^/deep-layer^more^ distribution pattern (Fig. 1f’). In the *Pdk1* cKO cortex, this distribution pattern seemed unchanged; however, the number of SST^+^ interneurons was obviously decreased ((Fig. 1g’). There was a 32% decrease compared to the control cortex (Fig. 1h). Furthermore, we observed a 67% decrease in the number of PV^+^ interneurons at P15 (Fig. 1i-k). As shown by PROX1 immunostaining, the control cortex displayed a superficial layer^more^/deep-layer^less^ distribution pattern of CGE-derived interneurons that was consistent with previously reported results. In the *Pdk1* cKO cortex, only a few PROX1^+^ interneurons were detected. The percentage was reduced to 36.2% compared to that of control mice (Fig. 1l-n). These results suggested that the number of cortical interneurons, including both MGE- and CGE-derived interneurons, was significantly decreased after *Pdk1* ablation.

### Embryonic cell loss in the cortex with unchanged tangential migration after *Pdk1* deletion

The migration of cortical interneurons is initiated as early as E12.5. Once exiting the cell cycle, newborn cortical interneurons tangentially migrate towards the dorsal cortex [43]. Considering the important role of PDK1 in the migration of cortical excitatory neurons and cancer cells, we next examined whether the reduction of cortical interneurons in the *Pdk1* cKO cortex was caused by defects of tangential migration. At E12.5, a small portion of GFP^+^ cortical interneurons in *Dlx5/6-Cre-EGFP; Pdk1*^*fl/+*^ control mice had already migrated across the pallial-subpallial boundary (PSB) and entered the dorsal cortex (Fig. 2a). The same result was observed in *Pdk1* cKO mice, and the distribution patterns and the numbers of GFP^+^ were comparable (Fig. 2b and t). Similar to the results observed at E12.5, more GFP^+^ cortical interneurons migrated into the developing cortical plate, and no significant differences were observed at E13.5 (Fig. 2c-d’ and 2 T). These results indicated that the number of newborn cortical interneurons was not altered at early stages. From E14.5 onwards, we observed that interneurons that in the cortical plate and in the migratory routes were gradually decreased (Fig. 2e-j’). At E14.5, this reduction seemed subtle (Fig. 2e and f), while at E16.5, the reduction became more obvious (Fig. 2g and h). Till E18.5 the number of cortical interneurons was significantly decreased (Fig. 2i-j’ and t). To further address whether *Pdk1* deletion had effects on tangential migration ability, we quantified the dorsal/ventral ratio of the number of GFP^+^ Interneurons at E13.5 and E18.5, respectively. No significant differences were detected between *Pdk1* cKO and control mice (Fig. 2c-d” and i-j” and u). Combined with unchanged numbers of GFP^+^ interneurons in the dorsal cortex at early stages, these results indicated that *Pdk1* deletion had no effects on tangential migration ability.

To further address whether *Pdk1* deletion affects distinct subtypes of cortical interneurons derived from the MGE and CGE, respectively, we performed in situ hybridization to detect SST and immunostaining to detect PROX1. As shown in Fig. 2k-p’, the number of MGE-derived SST^+^ interneurons in the *Pdk1* cKO subpallium was obviously decreased from E14.5 onwards. At E18.5, the number of SST^+^ interneurons was further reduced in the *Pdk1* cKO cortex (Fig. 2o-p’ and v). Notably, no SST^+^ interneurons accumulated in the ventral telencephalon (Fig. 2k-p), further demonstrating that *Pdk1* is not required for the tangential migration. Next, we determined the number of CGE-derived PROX1^+^ interneurons in the cortical plate at E18.5, and similar to SST^+^ interneurons, a significant decrease was observed (Fig. 2q-r’ and s). Taken together, these results show that cortical interneuron loss occurred at early embryonic stages but *Pdk1* ablation had no obvious effects on the tangential migration.

### The progenitor Pool in the ventral telencephalon was not altered

**Fig. 3:**
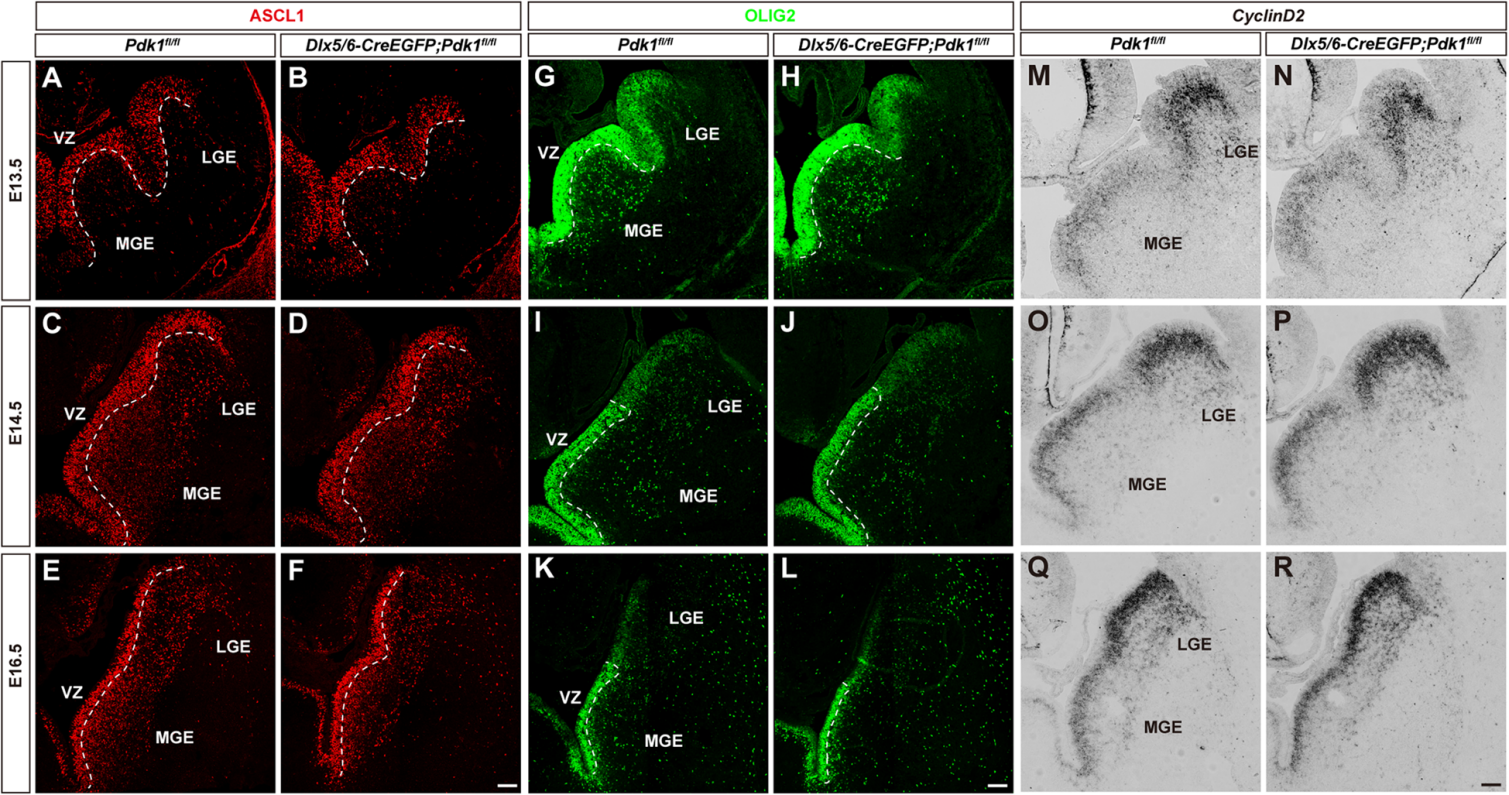
The progenitor pool in the ventral telencephalon was not altered. (**a-f**) Immunofluorescence for ASCL1 in coronal sections showed that the ASCL1^+^ progenitor pool in the VZ was unaffected from E12.5 to E16.5. (**g-l**) Immunofluorescence for OLIG2 showed a comparable number of OLIG2^+^ progenitors between control and *Pdk1* cKO mice from E12.5 to E16.5. (**m-r**) In situ hybridization for *CyclinD2* showed that the SVZ progenitor pool in the *Pdk1* cKO subpallium was normal from E12.5 to E16.5. Scale bar, 100 μm

Previously, we have showed that loss of PDK1 leads to a remarkable expansion of progenitor pools in the dorsal cortex and that PDK1 controls the transition from apical progenitors to basal progenitors during cortical excitatory neurogenesis [31]. To elucidate whether PDK1 plays a similar role in the development of interneurons, we examined progenitor pools in the ventral telencephalon. The transcription factor *Ascl1* is highly expressed in a subset of progenitors in the VZ and gradually down-regulated in the SVZ in the developing ventral telencephalon [44]. Loss of *Pdk1* has been shown to lead to a decreased level of *Ascl1* and cause an abnormal differentiation of interneurons [36]. *Ascl1* mutant mice exhibit a severe loss of progenitors, particularly in the MGE [45]. Here, no obvious differences in progenitors were observed in *Pdk1* cKO mice at E13.5 (Fig. 3a and b). Similar results were obtained at E14.5 (Fig. 3c and d) and E16.5 (Fig. 3e and f). These results indicated that loss of *Pdk1* had no effects on the ASCL1 ^+^ progenitor pool in the ventral telencephalon.

*Olig2* is expressed in most progenitors in the VZ and particularly strongly expressed in the MGE [46, 47]. OLIG2-expressing progenitors give rise to both cortical interneurons and oligodendrocytes [47, 48]. At E13.5, there were no significant differences in the GE between *Pdk1* cKO and control mice (Fig. 3g and h). Similarly, the staining was comparable at E14.5 (Fig. 3i and j) and E16.5 (Fig. 3k and l), suggesting an unchanged VZ progenitor pool.

Next, we examined the SVZ progenitor pool. Previous studies have demonstrated that *CyclinD2* is strongly expressed in intermediate progenitor cells in the SVZ of the ventral telencephalon [49, 50]. *CyclinD2* promotes transit-amplifying divisions in the SVZ and ensures the proper output of at least a subset of PV^+^ interneurons [51]. We did not find significant differences in *CyclinD2*-expressing progenitors in *Pdk1* cKO mice (Fig. 3m-r), suggesting that the intermediate progenitor pool was normal. Taken together, these results show that in contrast to the role of PDK1 in the dorsal cortex, loss of *Pdk1* had no effects on progenitor pools in the developing ventral telencephalon.

### Normal cell proliferation in the ventral telencephalon of *Pdk1* cKO mice

**Fig. 4:**
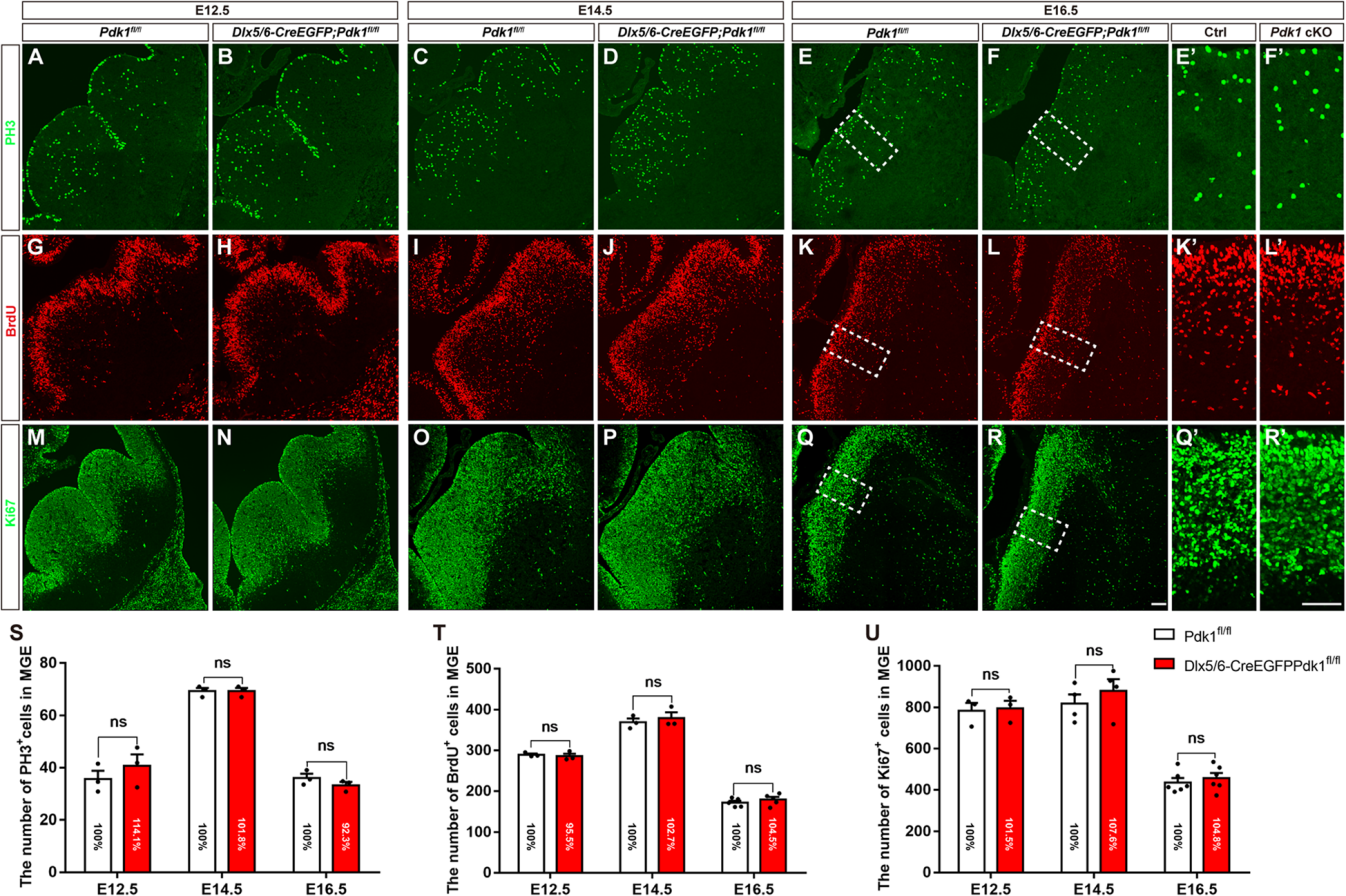
Normal cell proliferation in the *Pdk1* cKO subpallium. (**a-f***′*) Immunofluorescence for PH3 showed that the number of PH3-labeled M phase cells was unaffected in the MGE of *Pdk1* cKO mice compared with control at E12.5, E14.5, and E16.5. (**s**) Statistical analysis of PH3^+^ cells (E12.5 *Pdk1* cKO: N = 3, Ctrl: N = 3, *P* = 0.7922; E14.5 *Pdk1* cKO: N = 3, Ctrl: N = 3, *P* > 0.9999; E16.5 *Pdk1* cKO: N = 3, Ctrl: N = 3, *P* = 0.5767, Multiple t-test, with alpha = 0.05/3 = 0.0167). (**g-l***’*) Immunofluorescence for BrdU showed that the number of S phase cells within the *Pdk1* cKO MGE was similar to that in the control MGE at E12.5, E14.5, and E16.5. (**t**) Statistical analysis of BrdU^+^ cells (E12.5 *Pdk1* cKO: N = 3, Ctrl: N = 3, *P* = 0.9502; E14.5 *Pdk1* cKO: N = 3, Ctrl: N = 3, *P* = 0.9265; E16.5 *Pdk1* cKO: *N* = 5, Ctrl: N = 5, *P* = 0.7189, Multiple t-test, with alpha = 0.05/3 = 0.0167). (**m-r’**) Immunofluorescence for Ki67 revealed comparable numbers of Ki67^+^ cells between *Pdk1* cKO and control mice at E12.5, E14.5, and E16.5. (**u**) Statistical analysis of Ki67^+^ cells (E12.5 *Pdk1* cKO: N = 3, Ctrl: N = 3, *P* = 0.9954; E14.5 *Pdk1* cKO: N = 4, Ctrl: N = 4, *P* = 0.8033; E16.5 *Pdk1* cKO: *N* = 6, Ctrl: N = 6, *P* = 0.9003, Multiple t-test, with alpha = 0.05/3 = 0.0167). The data are presented as the mean ± SEM. Scale bar, 100 μm

PDK1 is highly expressed in cancer cells and promotes oncogenesis by regulating proliferation and survival [34]. Inhibition of the PDK1/AKT/GSK3 pathway causes cell proliferation defects by inducing cell cycle arrest (at G0/G1 and G2/M phases) [52–54]. We next evaluated whether cell proliferation contributes to the reduction in cortical interneurons observed in *Pdk1* cKO mice. The generation of MGE-derived SST^+^ interneurons peaks at E12.5, with PV^+^ interneurons peaking at E14.5 [55–57], while GCE-derived interneuron production appears to peaks at E16.5 [13, 58]. Thus, three time points, E12.5, E14.5, and E16.5, were chosen to assess cell proliferation. Immunostaining for phospho-histone-3 (PH3) was carried out to quantify progenitors in M phase of the cell cycle. As shown in Fig. 4a and b, at E12.5 PH3^+^ progenitors were distributed in the VZ and SVZ of the GE, and no obvious differences were observed. Cell counting showed that total numbers of PH3^+^ cells in the VZ and SVZ of the MGE were comparable (Fig. 4s). Similar results were obtained at E14.5 (Fig. 4c-d, and s) and E16.5 (Fig. 4e-f’, and s). We also measured progenitors in S phase by acute BrdU labeling. No significant differences were detected at E12.5 (Fig. 4g-h, and t), E14.5 (Fig. 4i-j, and t), or E16.5 (Fig. 4k-l’, and t). To further confirm the proliferation of progenitors, we next performed immunostaining of anti- Ki67. Consistent with the results of PH3 staining and BrdU-labeling, no obvious differences were identified between control and *Pdk1* cKO mice (Fig. 4m-r’ and u). Taken together, these results indicated that a lack of *Pdk1* has no effects on the proliferation of progenitors in the ventral telencephalon during cortical interneuron neurogenesis.

### PDK1 regulates the survival of the developing cortical interneurons

**Fig. 5:**
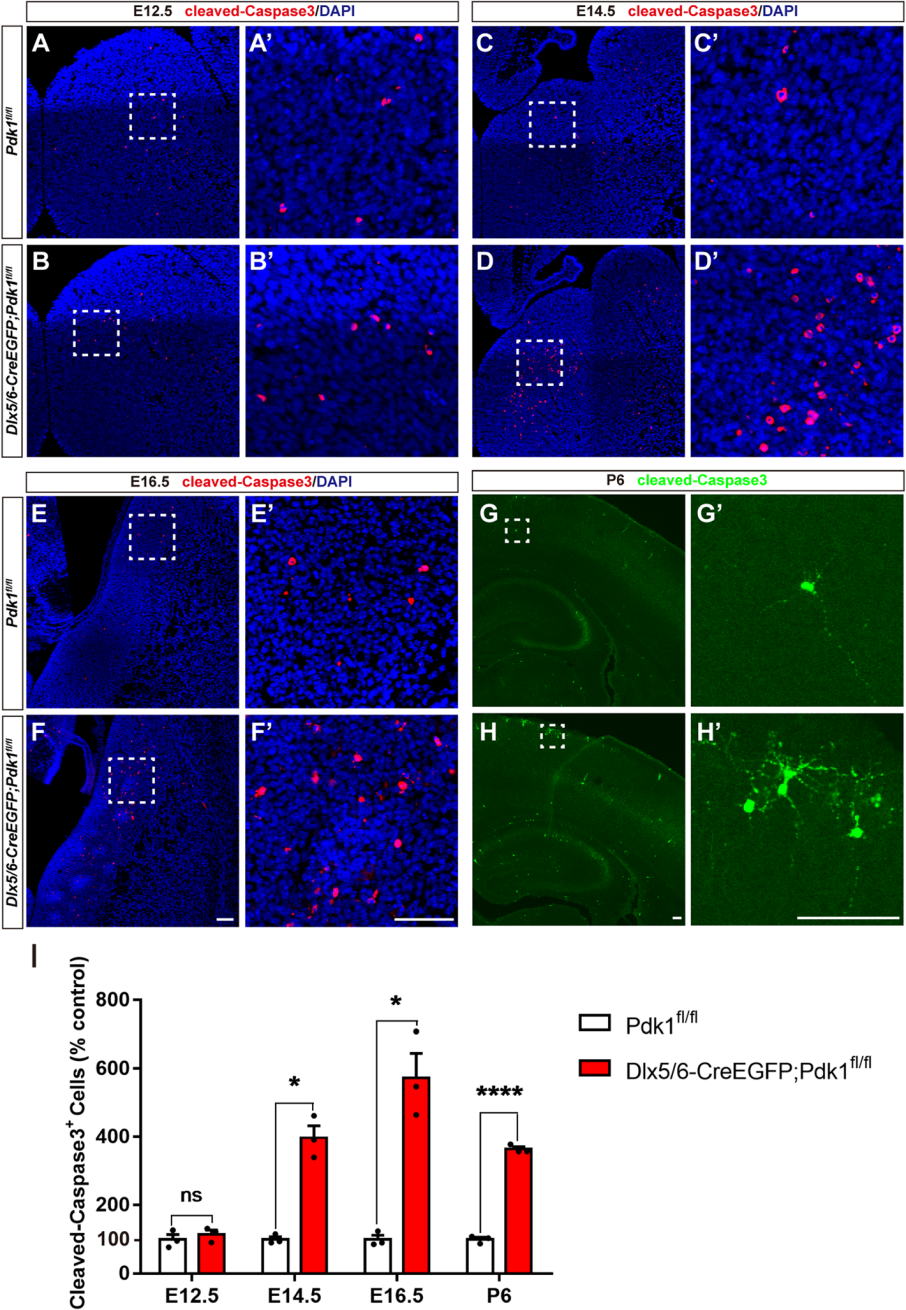
The loss of *Pdk1* resulted in increased cell apoptosis. (**a-b***′*) Immunofluorescence showing that the number of Caspase-3^+^ cells was no differences in the subpallium of *Pdk1* cKO and control mice at E12.5. (**c-d’**) The number of Caspase-3^+^ cells was significantly increased in the *Pdk1* cKO subpallium compared with control at E14.5. (**e-f***′*) Increased number of Caspase-3^+^ cells in the *Pdk1* cKO subpallium compared to control at E16.5. (**g-h***′*) Immunofluorescence for Caspase-3 showed that the number of Caspase-3^+^ cells was increased in the cortex of *Pdk1* cKO mice compared with control mice at P6. (**i**) Statistical analysis of Caspase-3^+^ cells (E12.5 *Pdk1* cKO: N = 3; Ctrl: N = 3; *P* = 0.9231; E14.5 *Pdk1* cKO: N = 3; Ctrl: N = 3; *P* = 0.0048; E16.5 *Pdk1* cKO: N = 3; Ctrl: N = 3; *P* = 0.0114; P6 *Pdk1* cKO: N = 3; Ctrl: N = 3; *P* = 0.00004, Multiple t-test, with alpha = 0.05/4 = 0.0125). The data are presented as the mean ± SEM. Scale bar, 100 μm

The unchanged tangential migration and cell proliferation in *Pdk1* cKO mice led us to further explore whether the reduction in cortical interneurons was due to increased apoptosis. To examine this hypothesis, we performed immunostaining for the cleaved (active) form of Caspase-3. At E12.5 in both control and *Pdk1* cKO mice, only a few Caspase-3^+^ cells were distributed in the GE (Fig. 5a-b’). Statistical analysis showed no remarkable differences (Fig. 5i), suggesting that cell apoptosis was not increased at this time point, coincident with the unchanged number of GFP^+^ interneurons at E12.5 (Fig. 2a-b and s). In contrast, we observed a significant increase in apoptosis in the GE at E14.5 (Fig. 5c-d’). Compared with control mice, the number of Caspase-3^+^ cells was increased by 297.3% (Fig. 5i). Similarly, at E16.5, the number of Caspase-3^+^ cells was significantly increased as well (Fig. 5e-f’ and 5I). We also observed a remarkable increase in cell apoptosis in the postnatal neocortex (Fig. 5g-h’ and i). These results thus demonstrated that *Pdk1* ablation resulted in severely increased apoptosis and subsequently led to a reduction in cortical interneurons. PDK1 plays a critical role in regulating the survival of cortical interneurons. Since *Pdk1* was deleted in the SVZ progenitors in the ventral telencephalon in this study, the possibility that apoptotic cells includes a very small number of progenitors in the SVZ can’t be excluded.

### Decreased activity of AKT-GSK3β signaling pathway after *Pdk1* deletion

**Fig. 6:**
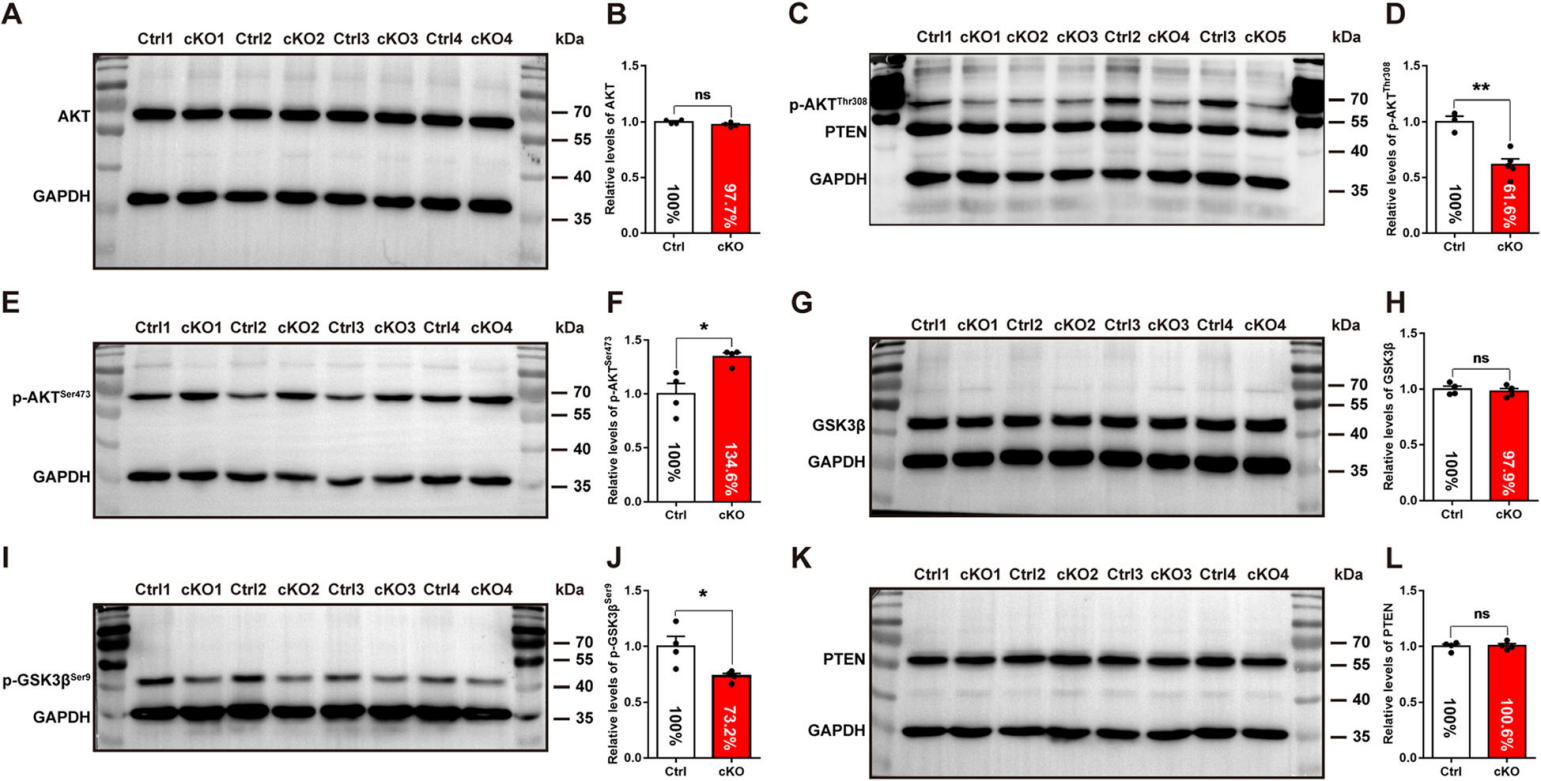
Decreased activity of AKT-GSK3β signaling pathway after *Pdk1* deletion. Western blotting analysis of the AKT-GSK3β signaling pathway in the subpallium at E16.5. (**a**) The relative expression levels of total AKT were no differences between *Pdk1* cKO and control mice. (**b**) Statistical analysis of total AKT protein expression levels (*Pdk1* cKO: N = 4; Ctrl: N = 4; *P* = 0.1186, Student’s t-test, with alpha = 0.05). (**c**) The relative expression levels of p-AKT^Thr308^ was significantly reduced. (**d**) Statistical analysis of p-AKT^Thr308^ protein expression levels (*Pdk1* cKO: N = 5; Ctrl: N = 3; *P* = 0.0027, Student’s t-test, with alpha = 0.05). (**e**) While p-AKT^Ser473^ was obviously increased in the *Pdk1* cKO subpallium compare to control. (**f**) Statistical analysis of p-AKT^Ser473^ protein expression levels (*Pdk1* cKO: N = 4; Ctrl: N = 4; *P* = 0.0153, Student’s t-test, with alpha = 0.05). (**g**) There were no significant differences in the relative expression levels of total GSK3β between *Pdk1* cKO and control mice. (**h**) Statistical analysis of total GSK3β protein expression levels (*Pdk1* cKO: N = 4; Ctrl: N = 4; *P* = 0.5900, Student’s t-test, with alpha = 0.05). (**i**) The relative expression levels of p-GSK3β^Ser9^ was significantly decreased in the *Pdk1* cKO subpallium compared to control. (**j**) Statistical analysis of p-GSK3β^Ser9^ protein expression levels (*Pdk1* cKO: N = 4; Ctrl: N = 4; *P* = 0.0273, Student’s t-test, with alpha = 0.05). (**k**) There were no significant differences in the relative expression levels of PTEN between *Pdk1* cKO and control mice. (**l**) Statistical analysis of PTEN protein expression levels (*Pdk1* cKO: N = 4; Ctrl: N = 4; *P* = 0.8366, Student’s t-test, with alpha = 0.05). GAPDH was used as an internal control

The PDK1-AKT pathway is important for cell survival [59–63]. Previous studies have demonstrated that the phosphorylation of AKT at Thr308 by PDK1 is essential for its activation [64, 65]. Loss of *Pdk1* results in decreased AKT activity, consequently leading to an increased apoptosis of excitatory neurons at postnatal rather than embryonic stages [30]. Here, we first examined total AKT level in the ventral telencephalon and observed no significant differences at E16.5 (Fig. 6a and b); however, the phosphorylation level of AKT at Thr308 (p-AKT^Thr308^) was significantly decreased (Fig. 6c and d), reflecting a decreased activity after *Pdk1* ablation. In contrast, the relative level of AKT phosphorylation at Ser473 (p-AKT^Ser473^) was increased (Fig. 6e and f), indicating a compensatory effect for the decreased level of p-AKT^Thr308^. Studies have shown that active GSK3β plays a proapoptosis role [52, 54, 65–67], and that GSK3β can be inactivated when it is phosphorylated at Ser9 by AKT [68, 69]. We found that although the total level of GSK3β was unchanged (Fig. 6g and h), the level of GSK3β phosphorylated at Ser9 (p-GSK3β^Ser9^) was significantly decreased (Fig. 6i and j), consistent with the decrease of p-AKT^Thr308^. Phosphatase and tensin homolog (PTEN) serves as an inhibitor of the PI3K-PDK1-AKT signaling [70, 71]. It has been reported that the loss of *Pten* results in the preferential apoptosis of SST^+^ interneurons [8]. However, we did not detect significant changes in PTEN after *Pdk1* ablation (Fig. 6k and l). In summary, these results indicated that the loss of *Pdk1* resulted in a decreased AKT activity accompanied by a decreased level of inactivated GSK3β^Ser9^, ultimately leading to an increase in total GSK3β activity. Thus, the AKT-GSK3β signaling pathway may be involved in the regulation of the survival of cortical interneurons.

## Discussion

The PDK1-AKT signaling pathway regulates a variety of biological processes, including cell proliferation, differentiation, cell survival, and cell metabolism, in many tissues [72]. By inactivating GSK3β through phosphorylation that subsequently stabilizing the protein level of CyclinD1, PDK1-AKT pathway regulates cell proliferation in mouse fibroblasts [72, 73]. In the olfactory bulb PDK1 promotes stem cells to differentiate into neurons and astrocytes [74]. In *Drosophila* PDK1-AKT pathway modulates the function of multiple regulatory proteins, such as S6K, resulting in stimulation of glucose uptake and storage, and moreover controls protein synthesis and storage of amino acids [21, 75, 76]. Our previous studies have shown that PDK1 plays essential roles in the development of the neocortex and the dentate gyrus. In the dorsal cortex, PDK1 regulates the transitional period between apical progenitors and basal progenitors through mediating asymmetric division, further controlling neuronal output [31]. In the developing dentate gyrus, PDK1 is required to maintain a balance between cell proliferation and neurogenesis [32]. PDK1 is also involved in the radial migration of cortical projection neurons [29]. Previously, it was shown that PDK1 promotes the differentiation of progenitors into GABAergic interneurons rather than excitatory neurons [36]. However, the detailed function of PDK1 during interneuron development remains unclear. In this study, we report that PDK1 is required for the survival of cortical interneurons but has no obvious effects on cell proliferation and tangential migration. Deletion of *Pdk1* in the subpallium leads to decreased AKT signaling activity, which may be involved in the survival of the developing cortical interneurons.

PDK1 serves as an antiapoptotic factor in a variety of cell types, such as cancer cells, endothelial cells, and skeletal muscle cells [28, 61, 62, 77–81]. Recently, it was reported that the ablation of *Pdk1* in the dorsal telencephalon leads to the increased apoptosis of cortical excitatory neurons at postnatal rather than embryonic stages [30, 31]. Here, we found that during cortical interneuron development apoptosis occurred in the embryonic stages and lasted to the postnatal stages, resulting in a dramatical reduction in cortical interneurons, including MGE-derived SST^+^ and PV^+^ interneurons and the CGE-derived PROX1^+^ subtypes. We further demonstrated that AKT activity was significantly decreased, as reflected by the level of p-AKT^Thr308^. Subsequently, the activity of GSK3β, a key downstream effector of the PDK1-AKT signaling pathway, was increased, as reflected by the decreased phosphorylation of GSK3β at Ser9. Studies have demonstrated that increased GSK3β activity enhances apoptosis [54, 66, 67]. Thus, the AKT-GSK3β signaling pathway may be involved in the regulation of the survival of interneurons.

By inhibiting the PI3K-PDK1-AKT signaling pathway, PTEN promotes apoptosis [82–86]. Previous studies have demonstrated that loss of *Pten* leads to the preferential apoptosis of SST^+^ interneurons, producing an abnormal PV^+^ interneuron/SST^+^ interneuron ratio in the cortex [8]. Here, we did not detect any changes in PTEN expression in the *Pdk1* cKO mice. This result indicated that PTEN is not involved in the increased apoptosis of cortical interneurons.

Cortical interneurons originate in the GE [10, 11, 13, 87–90]. Inhibition of the PDK1/AKT/GSK3 pathway reduces cell proliferation defects by inducing cell cycle arrest [52–54]. *Pdk1* ablation leads to expansion of the progenitor pool in the dorsal cortex [31]. In this study, no alteration in progenitor pools was observed. Immunostaining to detect ASCL1 and OLIG2 showed comparable ASCL1 and OLIG2 expression patterns in control and the *Pdk1* cKO mice, and the numbers of progenitors in the VZ and SVZ were similar. In situ analysis of *CyclinD2* further revealed an unaltered SVZ progenitor pool. Our data suggested that PDK1 is not involved in the transition of apical progenitors to basal progenitors in the ventral telencephalon. Moreover, BrdU labeling and immunostaining to detect PH3 and Ki67 demonstrated that the loss of *Pdk1* had no effects on cell proliferation. Gotoh et al. have shown that deletion of *Pdk1* in VZ progenitors achieved by *Nestin-Cre* mediated recombination leads to abnormal differentiation of interneurons alone with a decreased level of Ascl1 [36], here we did not observed alteration of Ascl1 expression when *Pdk1* was deleted in SVZ progenitors, suggesting PDK1 may play different roles in distinct progenitor pools. Future studies will explore molecular mechanisms underlying the function of PDK1 in distinct progenitor populations. Previously studies have demonstrated that PDK1 regulates the radial migration of excitatory neurons by controlling the microtubule stabilization [29]. Here, we found that the tangential migration of cortical interneurons was normal after *Pdk1* disruption. These results suggest that the roles of PDK1 in the excitatory neurons and interneurons are quite different. Future studies will explore more detailed mechanisms underlying PDK1-mediated regulation of cell migration, proliferation and survival in neurodevelopment.

## Supplementary information

**Additional file 1.** Statistical results of raw data.

**Additional file 2.** Raw data supplementary materials

## Data Availability

The data generated or analyzed during this study are included in this published article.
