# Supplementary material for: PDK1 regulates the survival of the developing cortical interneurons
Source: Mol Brain. 2020 May 4;13:65. doi: 10.1186/s13041-020-00604-6 (PMC7197138; doi:10.1186/s13041-020-00604-6)

Raw Data Supplementary Materials

Figure 1A Raw Data. Relative mRNA level of *Pdk1* in the *Pdk1* cKO subpallium at E16.5. Quantified data was listed in Raw Data Excel sheet1. 3 pair brains from 3 different litters were analyzed. *P*=0.0004.

Figure 1C-E Raw Data. Immunostaining for GFP showed that the total number of cortical interneurons in the *Pdk1* cKO cortex was decreased at P6. 4 pair brains from 3 different litters were used for statistical analysis. Quantified data was listed in Raw Data Excel sheet2. *P*=0.0013. Scale bar, 100 µm.

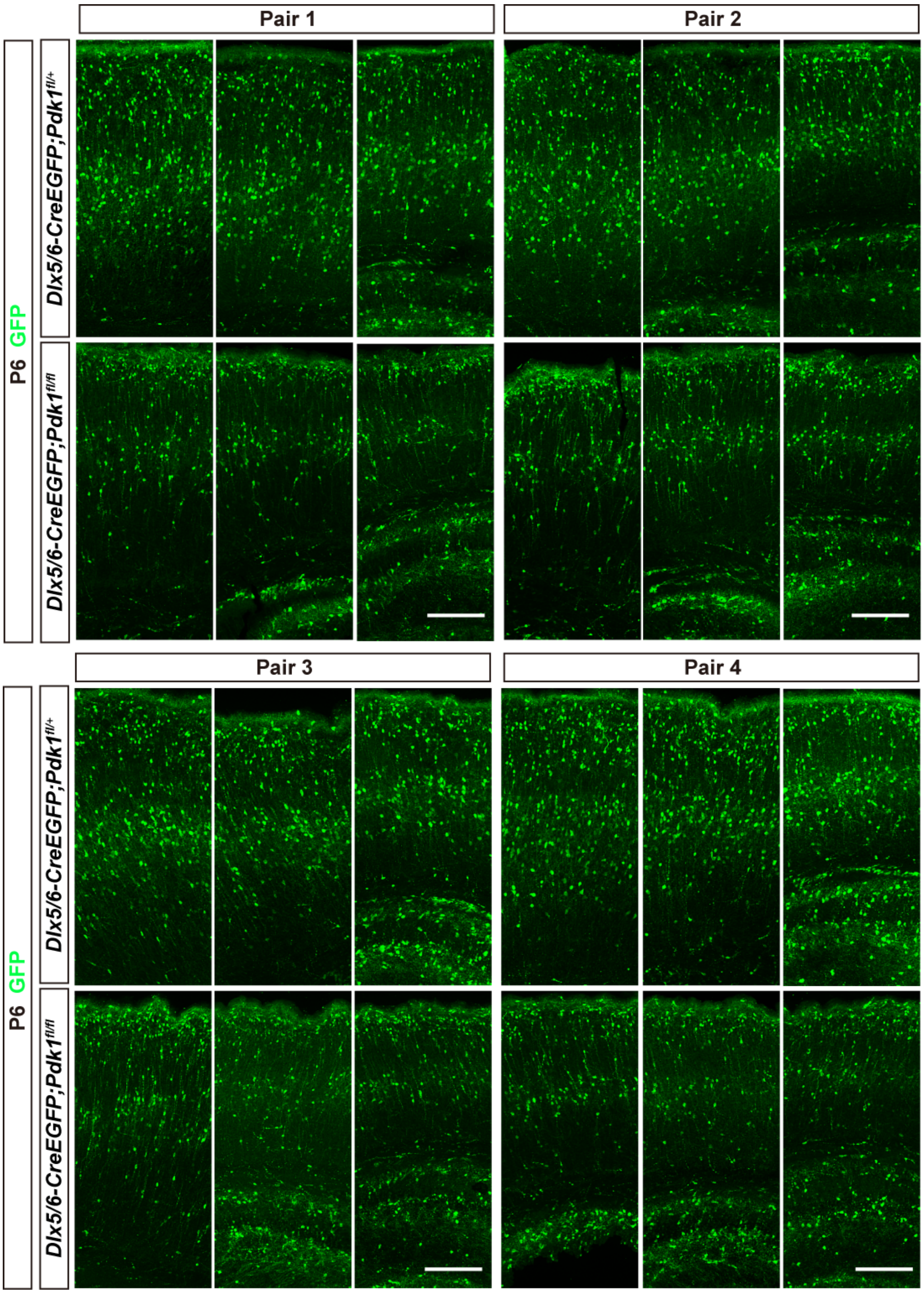

Figure 1F-H Raw Data. In situ hybridization of SST showed a reduction in SST^{+} interneurons in the *Pdk1* cKO cortex at P6. 3 pair brains from 3 different litters were used for statistical analysis. Quantified data was listed in Raw Data Excel sheet3. *P*=0.0230. Scale bar, 100 μm.

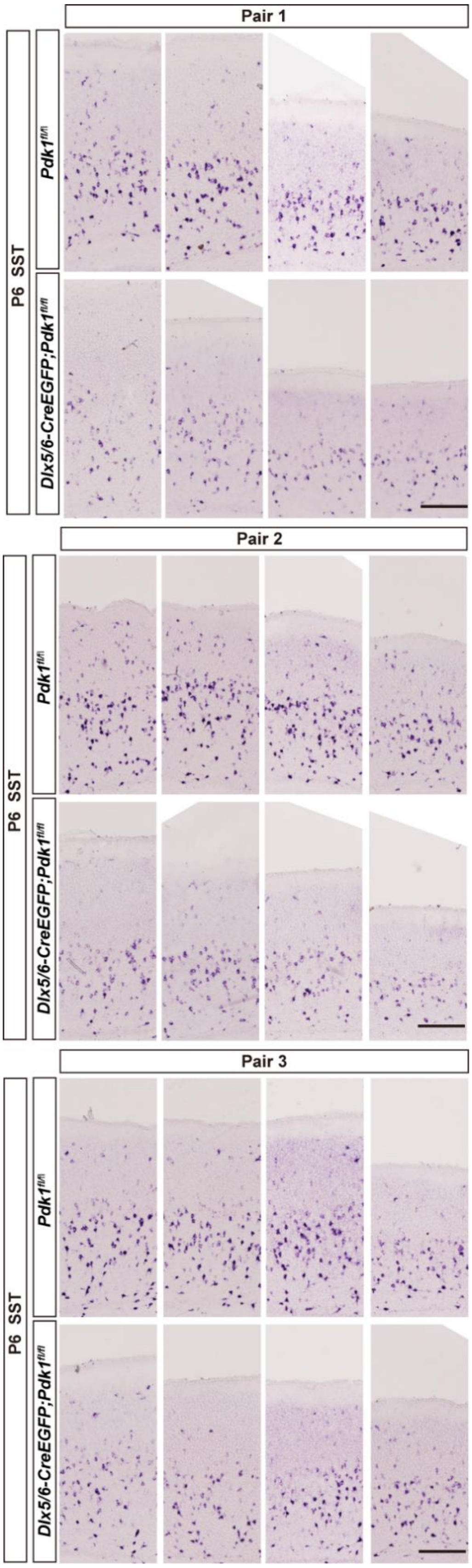

Figure 1I-K Raw Data. In situ hybridization of PV showed a reduction in PV+ interneurons in the *Pdk1* cKO cortex at P15. 4 pair brains from 3 different litters were used for statistical analysis. Quantified data was listed in Raw Data Excel sheet4. *P*=0.0013. Scale bar, 100 μm.

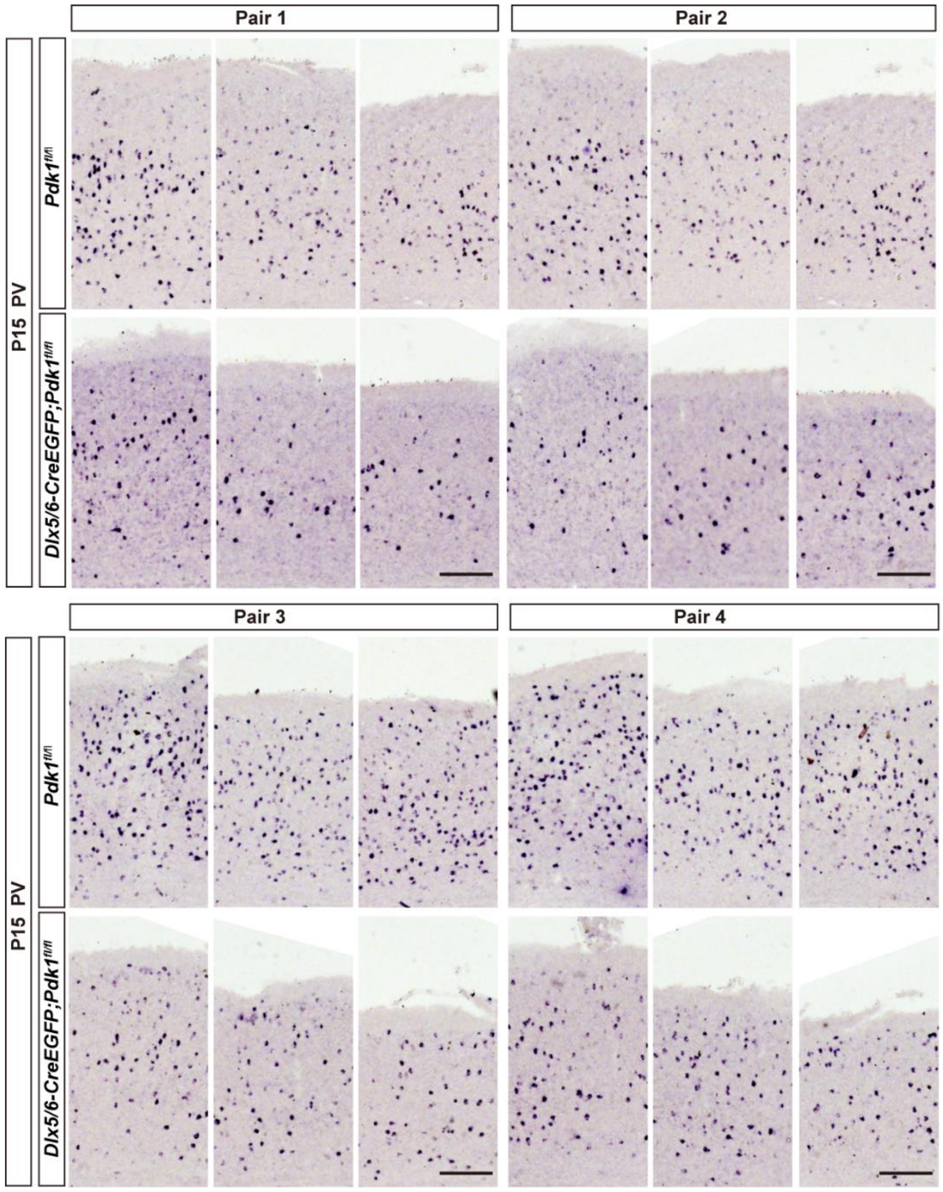

Figure 1L-N Raw Data. Immunostaining for PROX1 revealed that the number of CGE-derived cortical interneurons in the *Pdk1* cKO cortex was decreased at P6. 3 pair brains from 3 different litters were used for statistical analysis. Quantified data was listed in Raw Data Excel sheet5. *P*=0.0013. Scale bar, 100 μm.

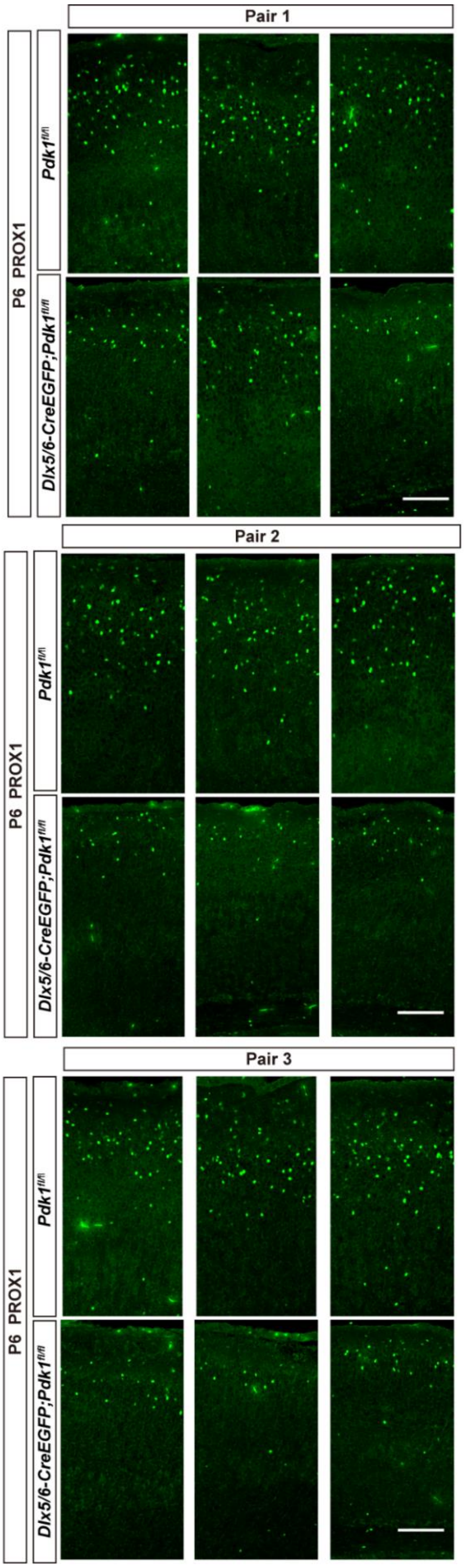

Figure 2A-B Raw Data. Immunofluorescence for GFP in coronal sections showed comparable distribution patterns and numbers of cortical interneurons at E12.5 between *Pdk1* cKO and control mice. 3 pair brains from 3 different litters were used for statistical analysis. Quantified data was listed in Raw Data Excel sheet6. P = 0.9912. Scale bar, 100 μm.

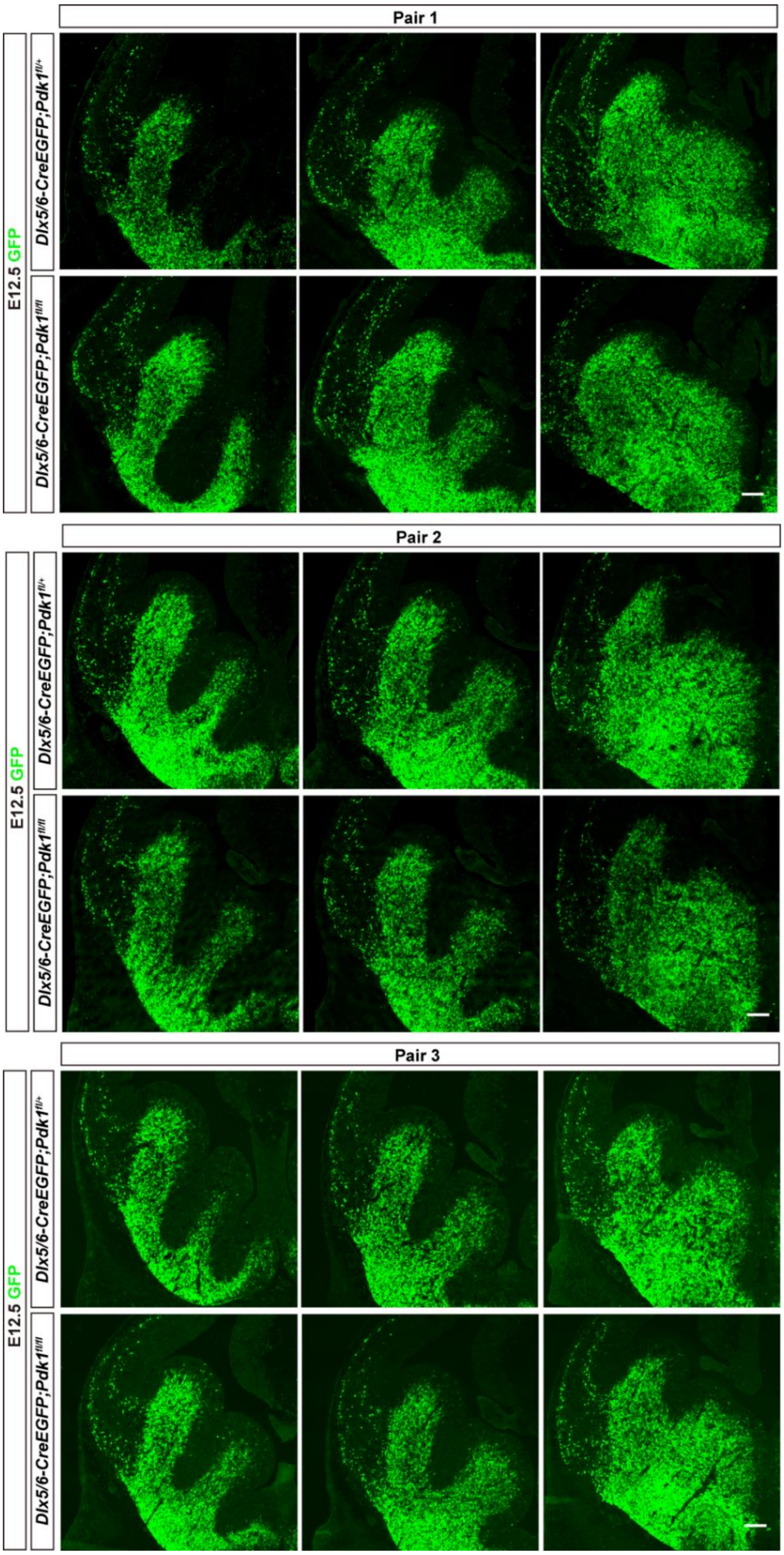

Figure 2C-D Raw Data. Immunofluorescence for GFP in coronal sections showed comparable distribution patterns and numbers of cortical interneurons at E13.5 between *Pdk1* cKO and control mice. 4 pair brains from 3 different litters were used for statistical analysis. Quantified data was listed in Raw Data Excel sheet6. *P* = 0.9089. Scale bar, 100 μm.

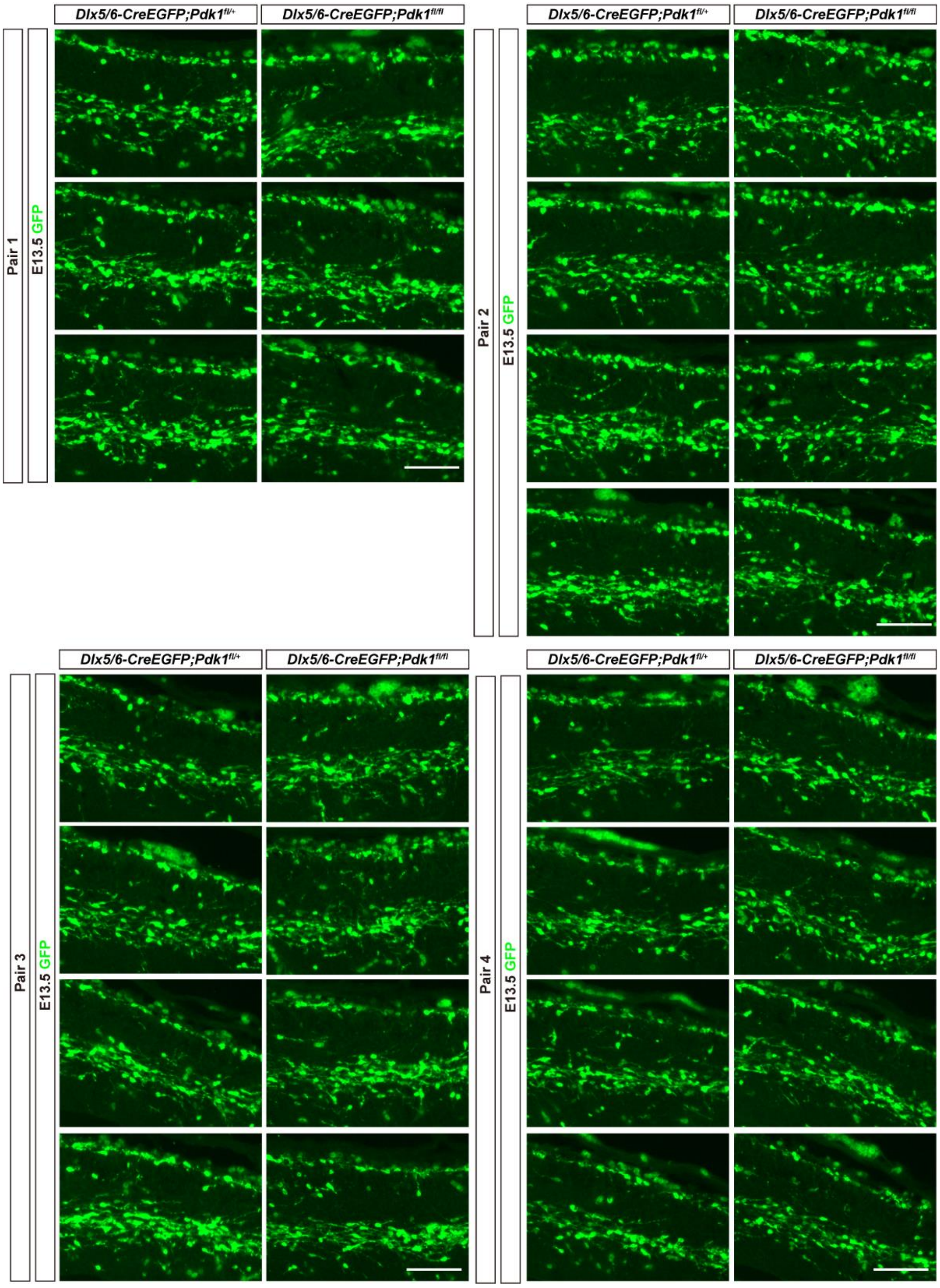

Figure 2C”-D” and 2U Raw Data. Raw Data. There were no significant differences in the dorsal/ventral ratio of numbers of interneurons between *Pdk1* cKO and control mice at E13.5. 4 pair brains from 3 different litters were used for statistical analysis. Quantified data was listed in Raw Data Excel sheet7. *P* = 0.1945.

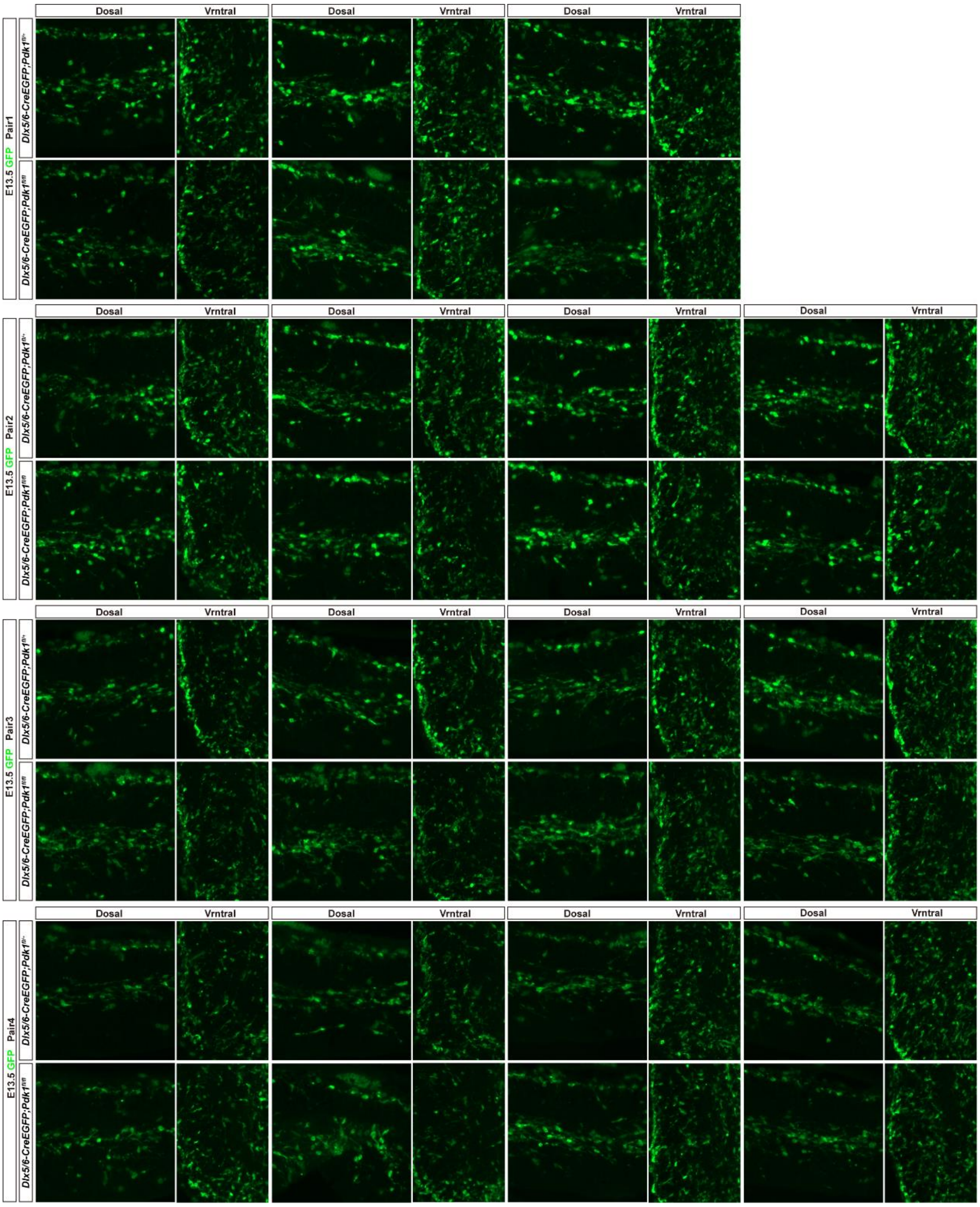

Figure 2I-J' Raw Data. The number of GFP^{+} cortical interneurons decreased at E18.5 in the *Pdk1* cKO cortex. 3 pair brains from 3 different litters were used for statistical analysis. Quantified data was listed in Raw Data Excel sheet6. *P* = 0.00034. Scale bar, 100 µm.

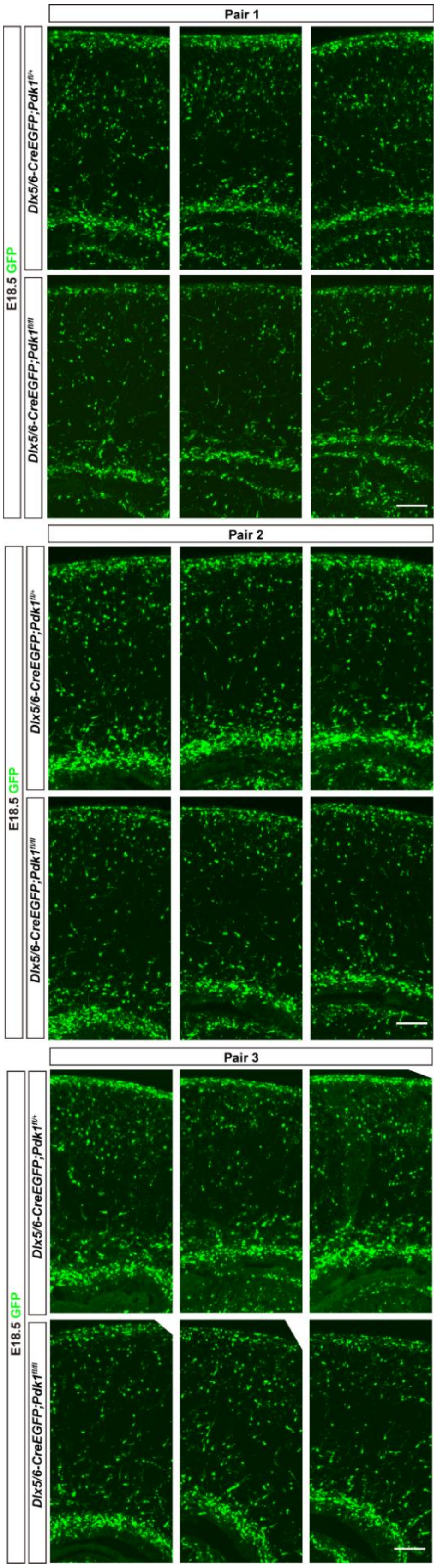

Figure 2I''-J'' and 2U Raw Data. There were no significant differences in the dorsal/ventral ratio of numbers of interneurons between *Pdk1* cKO and control mice at E18.5. 3 pair brains from 3 different litters were used for statistical analysis. Quantified data was listed in Raw Data Excel sheet7. P = 0.5371.

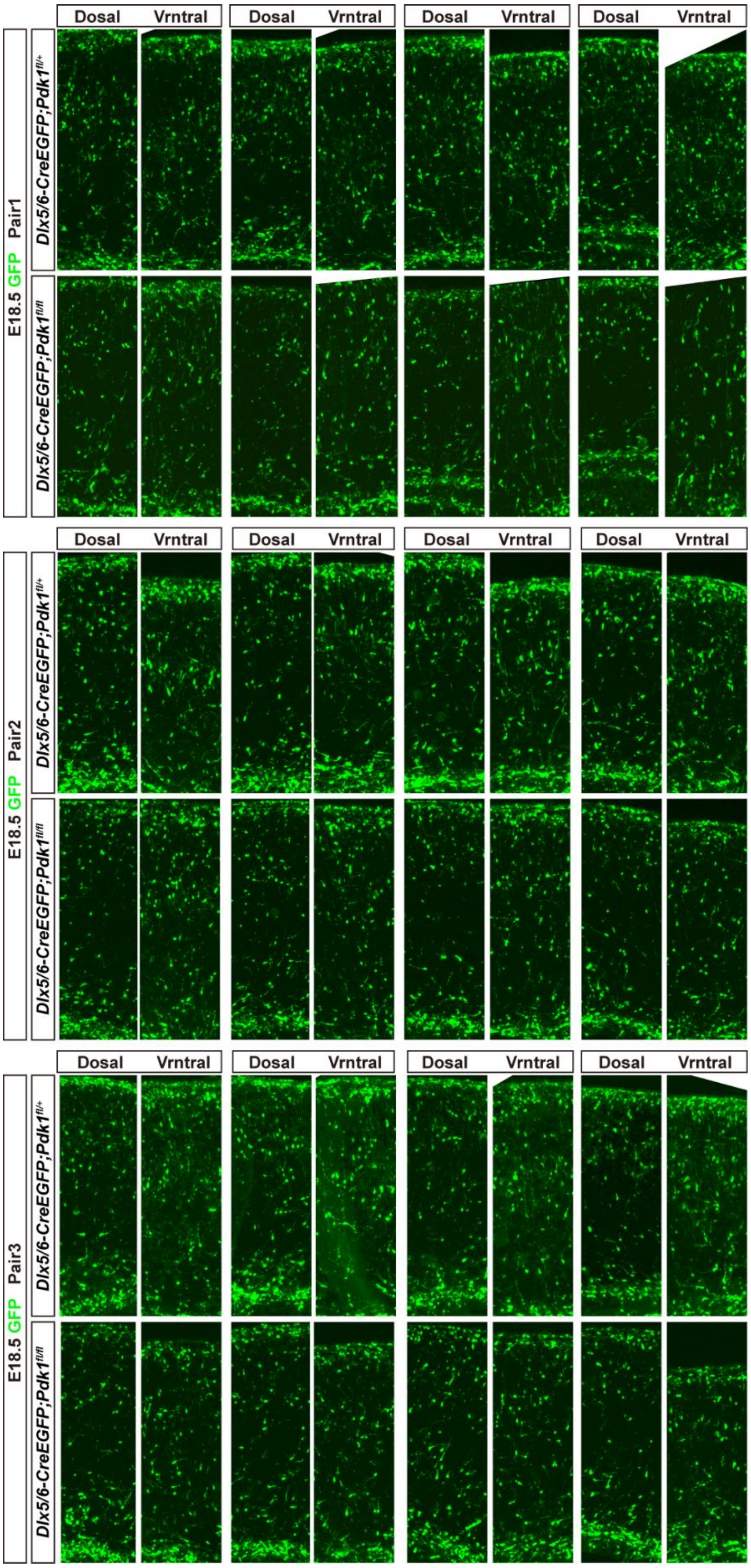

Figure 2K-L' Raw Data. In situ hybridization showed that the number of SST^{+} interneurons decreased at E14.5 in the *Pdk1* cKO subpallium. 3 pair brains from 3 different litters were used for statistical analysis. Quantified data was listed in Raw Data Excel sheet8. P = 0.00045. Scale bar, 100 μm.

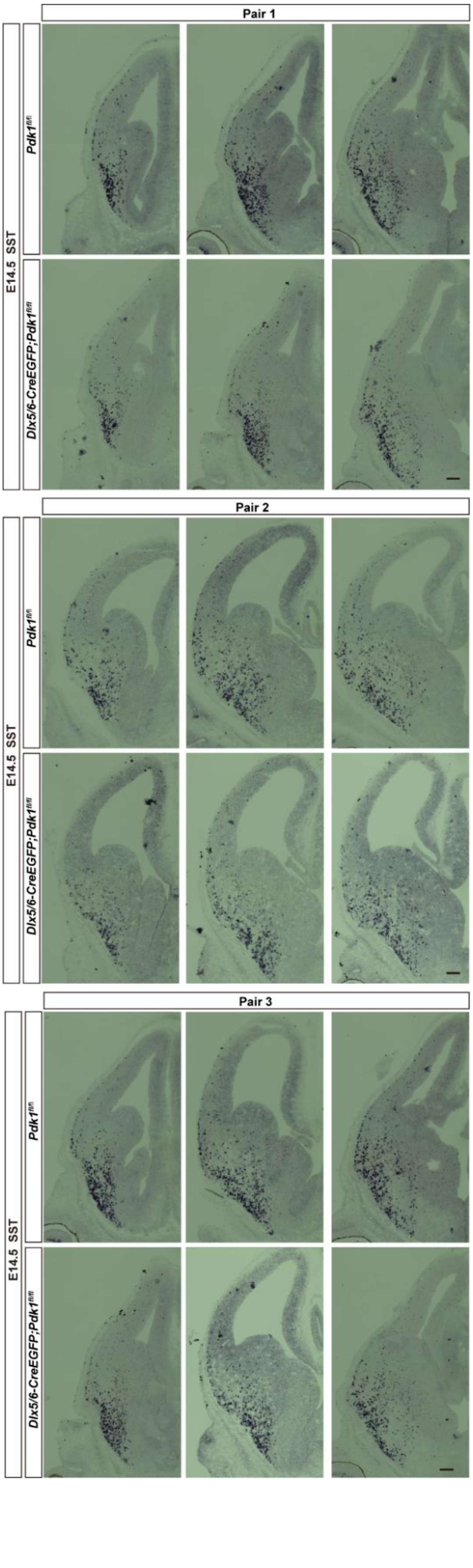

Figure 2M-N Raw Data. The number of SST^+ interneurons in the *Pdk1* cKO was decreased at E16.5. 3 pair brains from 3 different litters were used for analysis.

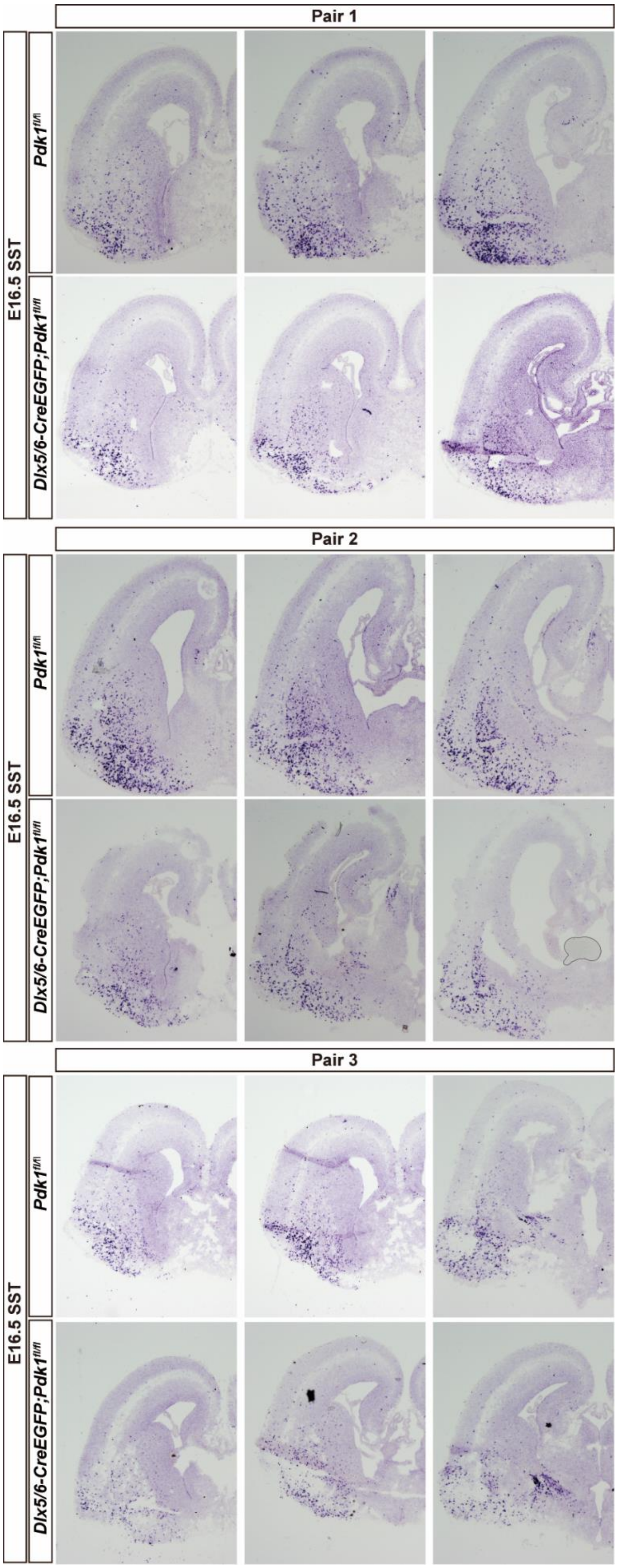

Figure 2O-P' Raw Data. The number of SST^{+} interneurons in the *Pdk1* cKO cortex was decreased at E18.5. 3 pair brains from 3 different litters were used for statistical analysis. Quantified data was listed in Raw Data Excel sheet8. *P* =0.00015. Scale bar, 100 μm.

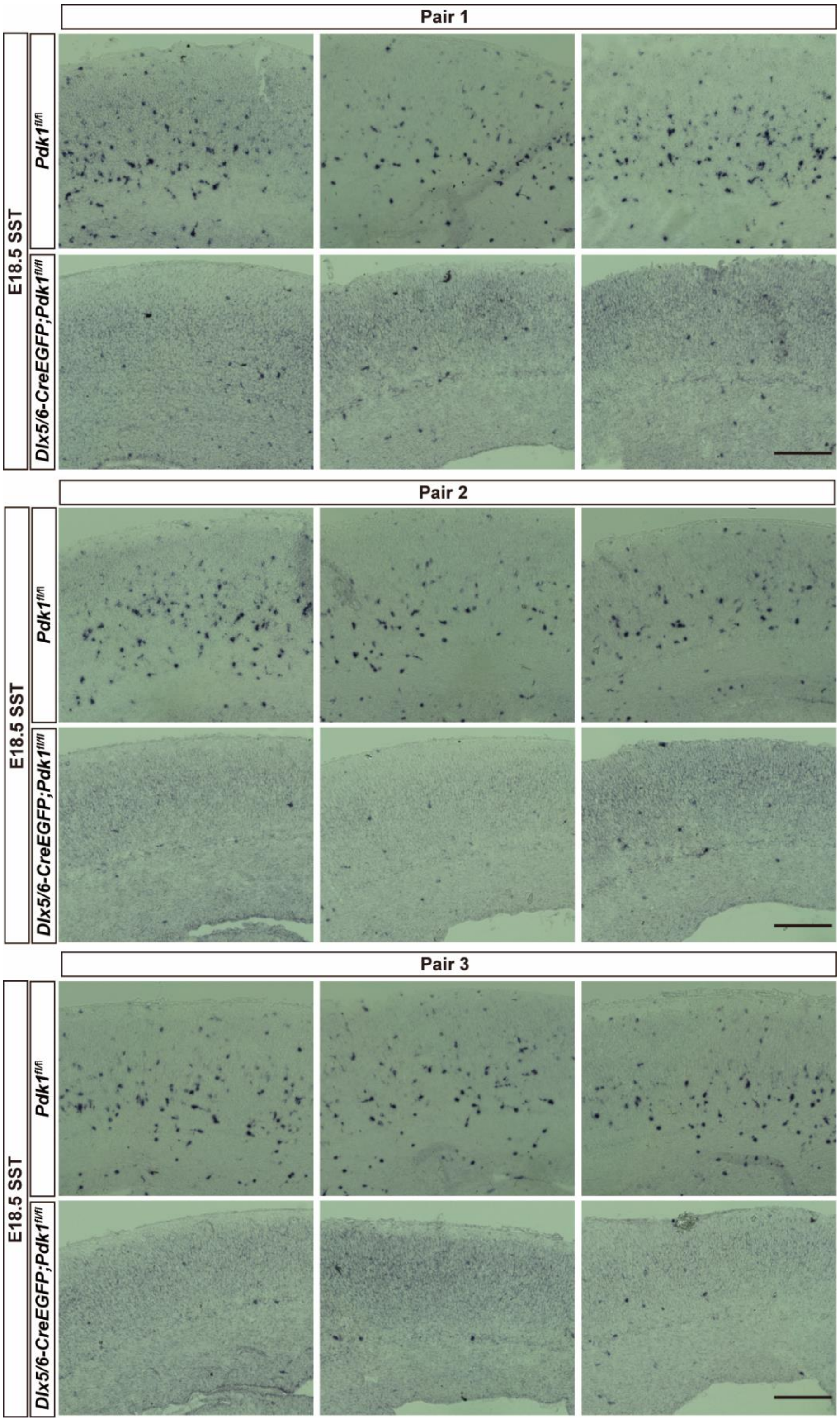

Figure 2Q-R' Raw Data. Immunofluorescence for PROX1 showed that the number of PROX1$^+$ cortical interneurons was decreased in the *Pdk1* cKO cortex at E18.5. 3 pair brains from 3 different litters were used for statistical analysis. Quantified data was listed in Raw Data Excel sheet9. P = 0.0018. Scale bar, 100 µm.

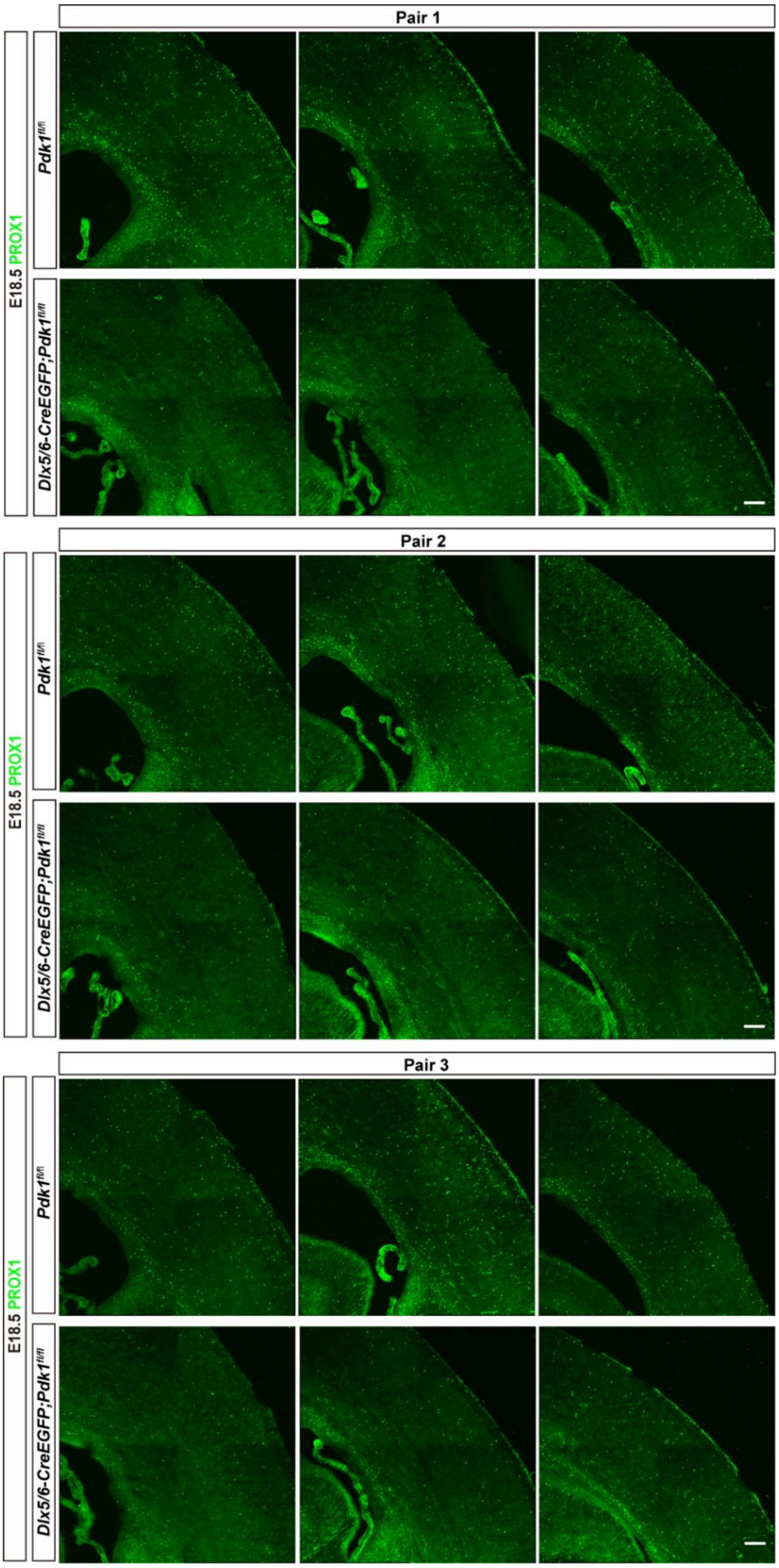

Figure 4A-B Raw Data. Immunofluorescence for PH3 showed that the number of PH3-labeled M phase cells was unaffected in the MGE of *Pdk1* cKO mice compared with control mice at E12.5. 3 pair brains from 3 different litters were used for statistical analysis. Quantified data was listed in Raw Data Excel sheet10. P = 0.7922. Scale bar, 100 µm.

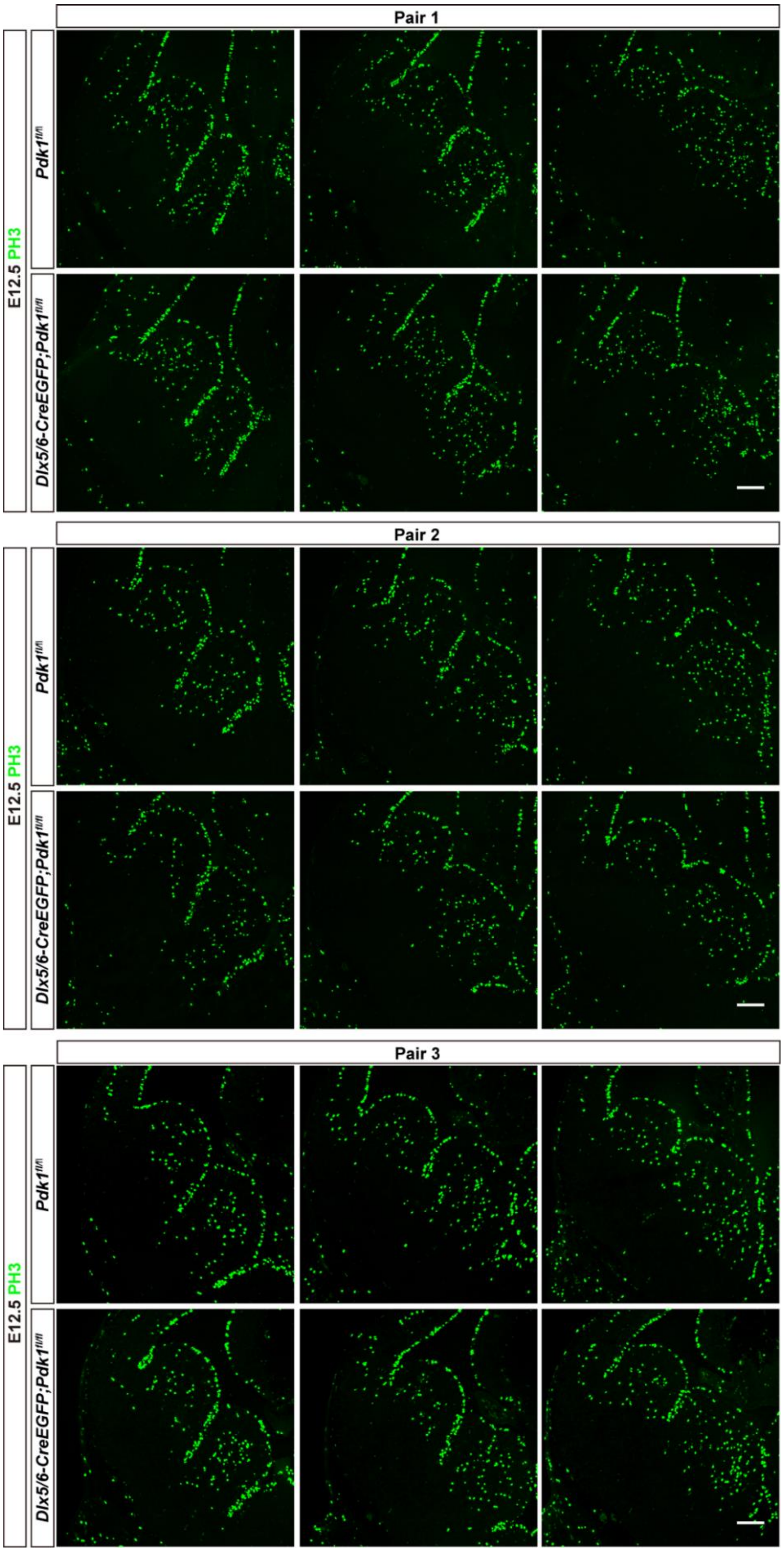

Figure 4C-D Raw Data. Immunofluorescence for PH3 showed that the number of PH3-labeled M phase cells was unaffected in the MGE of *Pdk1* cKO mice compared with control mice at E14.5. 3 pair brains from 3 different litters were used for statistical analysis. Quantified data was listed in Raw Data Excel sheet10. P >0.99999. Scale bar, 100 μm.

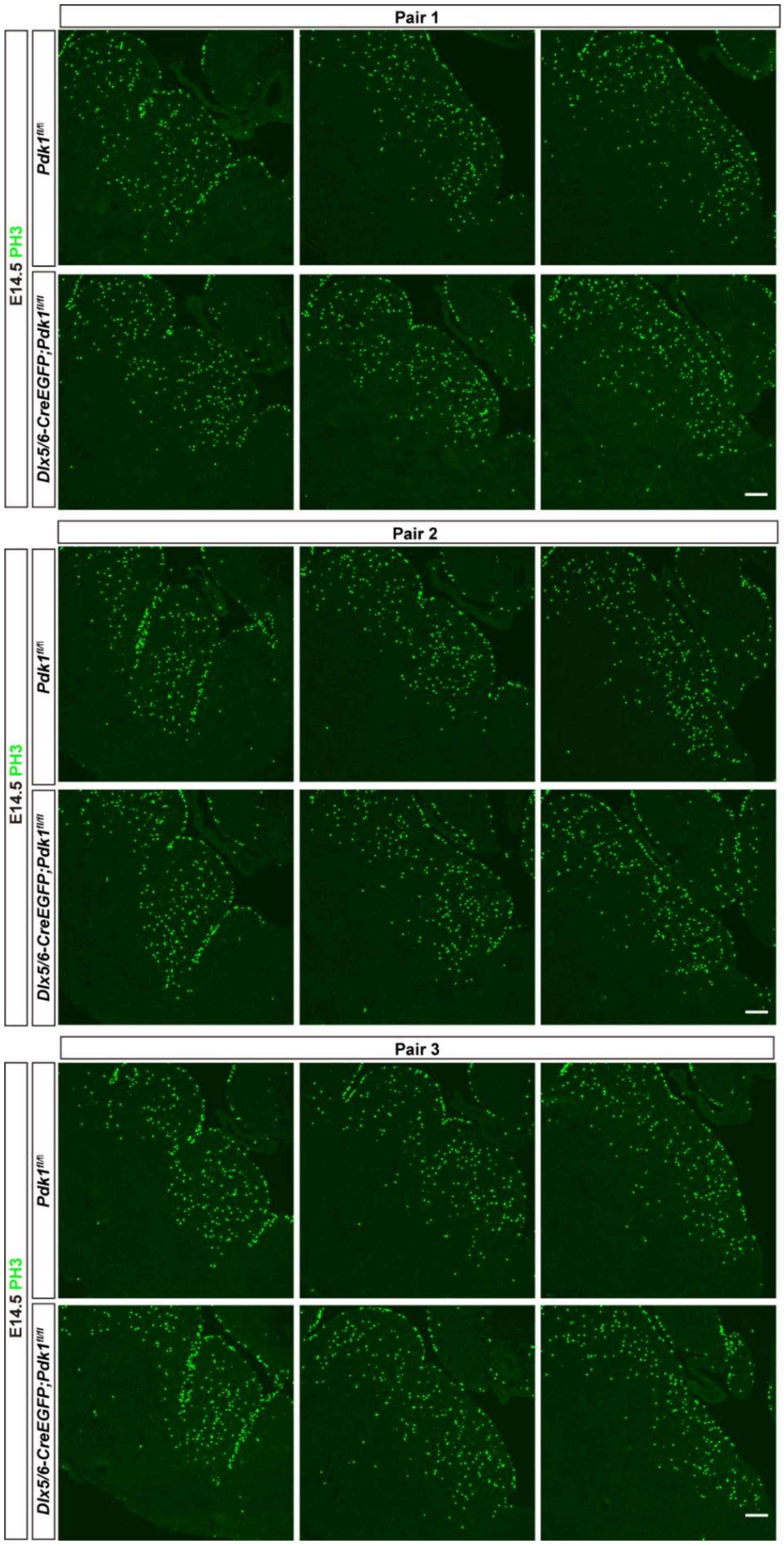

Figure 4E-F Raw Data. Immunofluorescence for PH3 showed that the number of PH3-labeled M phase cells was unaffected in the MGE of *Pdk1* cKO mice compared with control mice at E16.5. 3 pair brains from 3 different litters were used for statistical analysis. Quantified data was listed in Raw Data Excel sheet10. P = 0.5767. Scale bar, 100 μm.

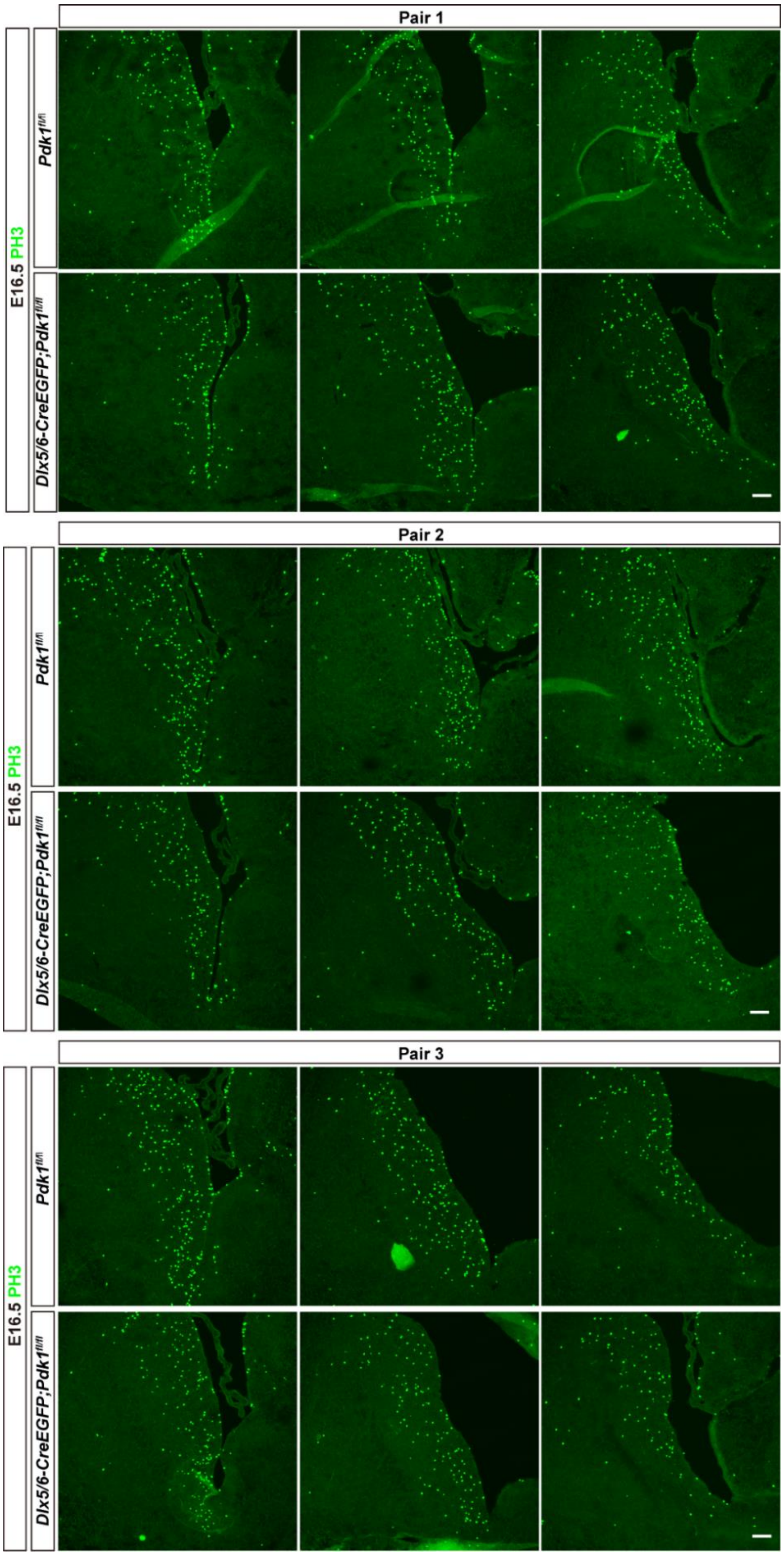

Figure 4G-H Raw Data. Immunofluorescence for BrdU showed that the number of S phase cells within the *Pdk1* cKO MGE was similar to that in the control MGE at E12.5. 3 pair brains from 3 different litters were used for statistical analysis. Quantified data was listed in Raw Data Excel sheet11. *P* = 0.9502. Scale bar, 100 μm.

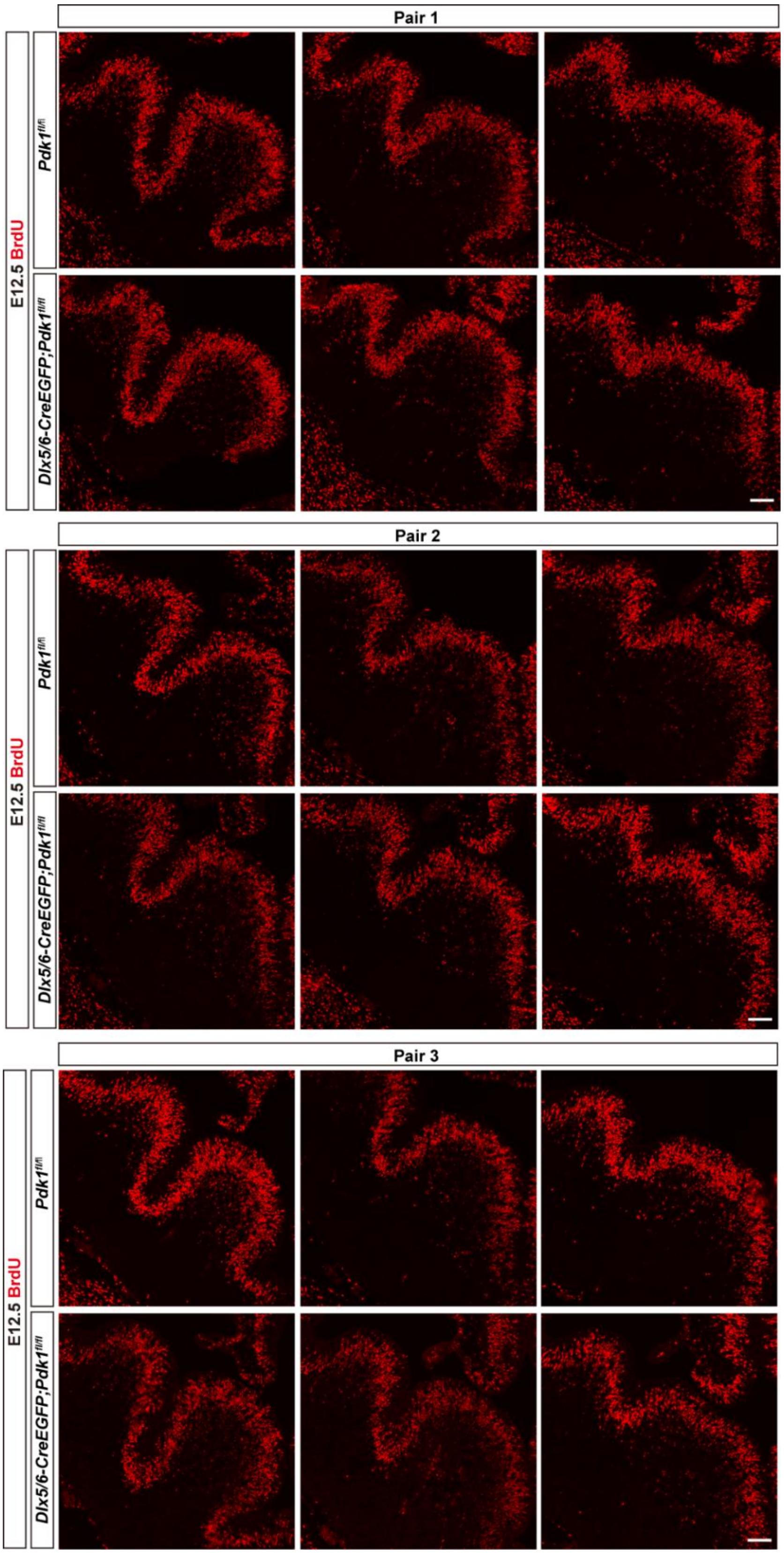

Figure 4I-J Raw Data. Immunofluorescence for BrdU showed that the number of S phase cells within the *Pdk1* cKO MGE was similar to that in the control MGE at E14.5. 3 pair brains from 3 different litters were used for statistical analysis. Quantified data was listed in Raw Data Excel sheet11. *P* = 0.9265. Scale bar, 100 μm.

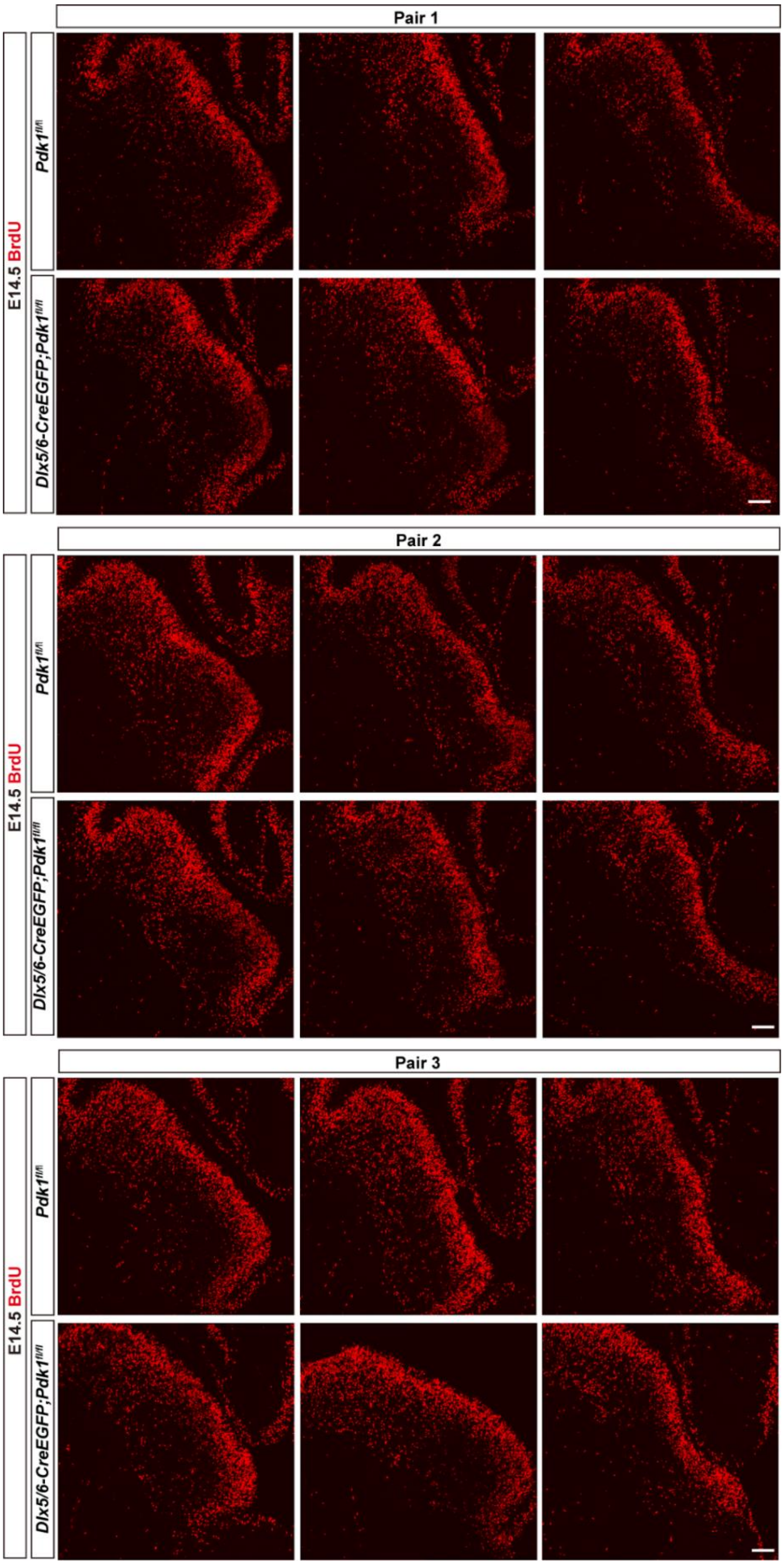

Figure 4K-L' Raw Data. Immunofluorescence for BrdU showed that the number of S phase cells within the *Pdk1* cKO MGE was similar to that in the control MGE at E16.5. 5 pair brains from 3 different litters were used for statistical analysis. Quantified data was listed in Raw Data Excel sheet11. P = 0.7189. Scale bar, 100 μm.

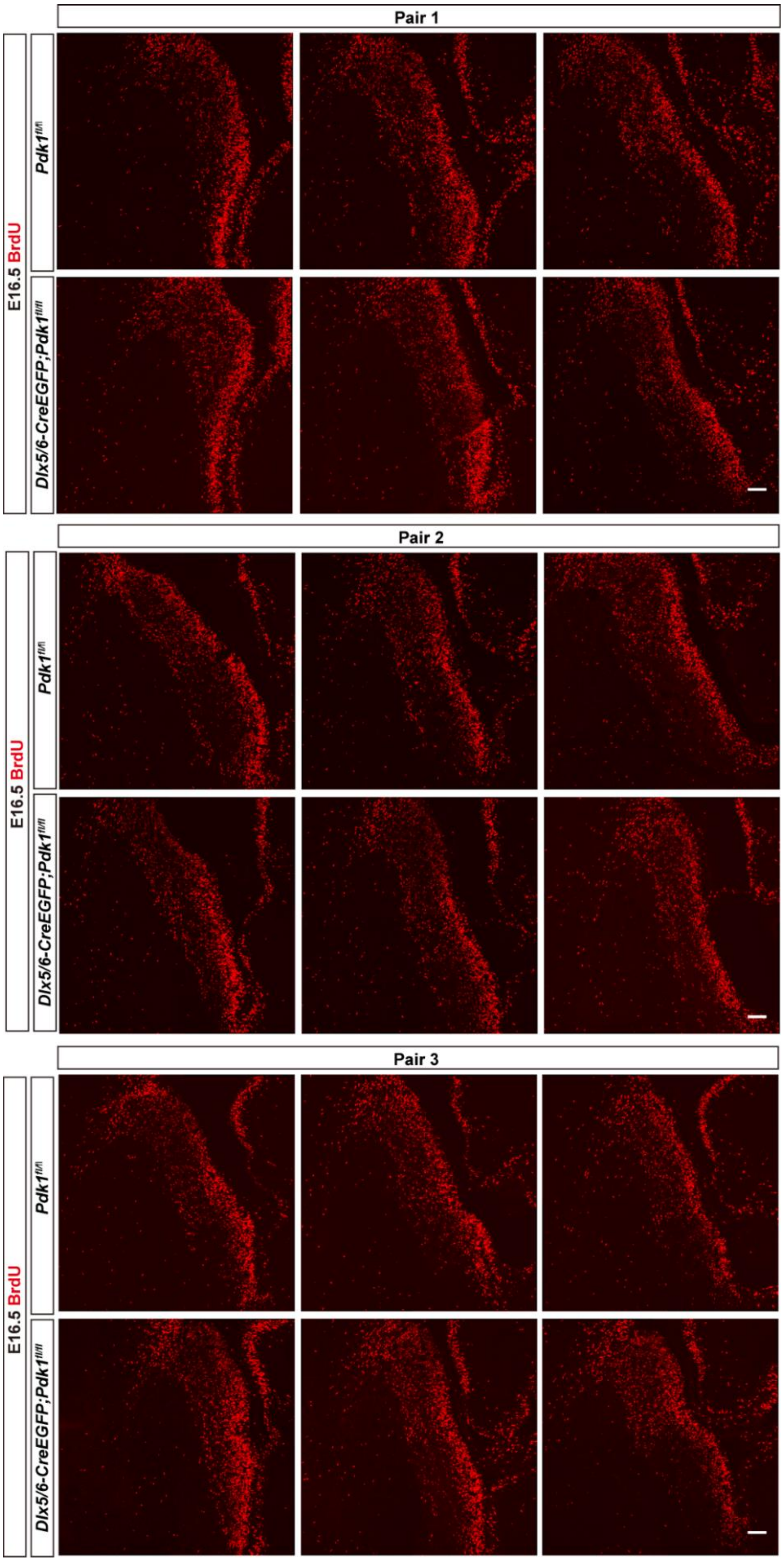

Figure 4M-N Raw Data. Immunofluorescence for Ki67 revealed comparable numbers of Ki67⁺ cells between *Pdk1* cKO and control mice at E12.5. 3 pair brains from 3 different litters were used for statistical analysis. Quantified data was listed in Raw Data Excel sheet12. *P* = 0.9954. Scale bar, 100 μm.

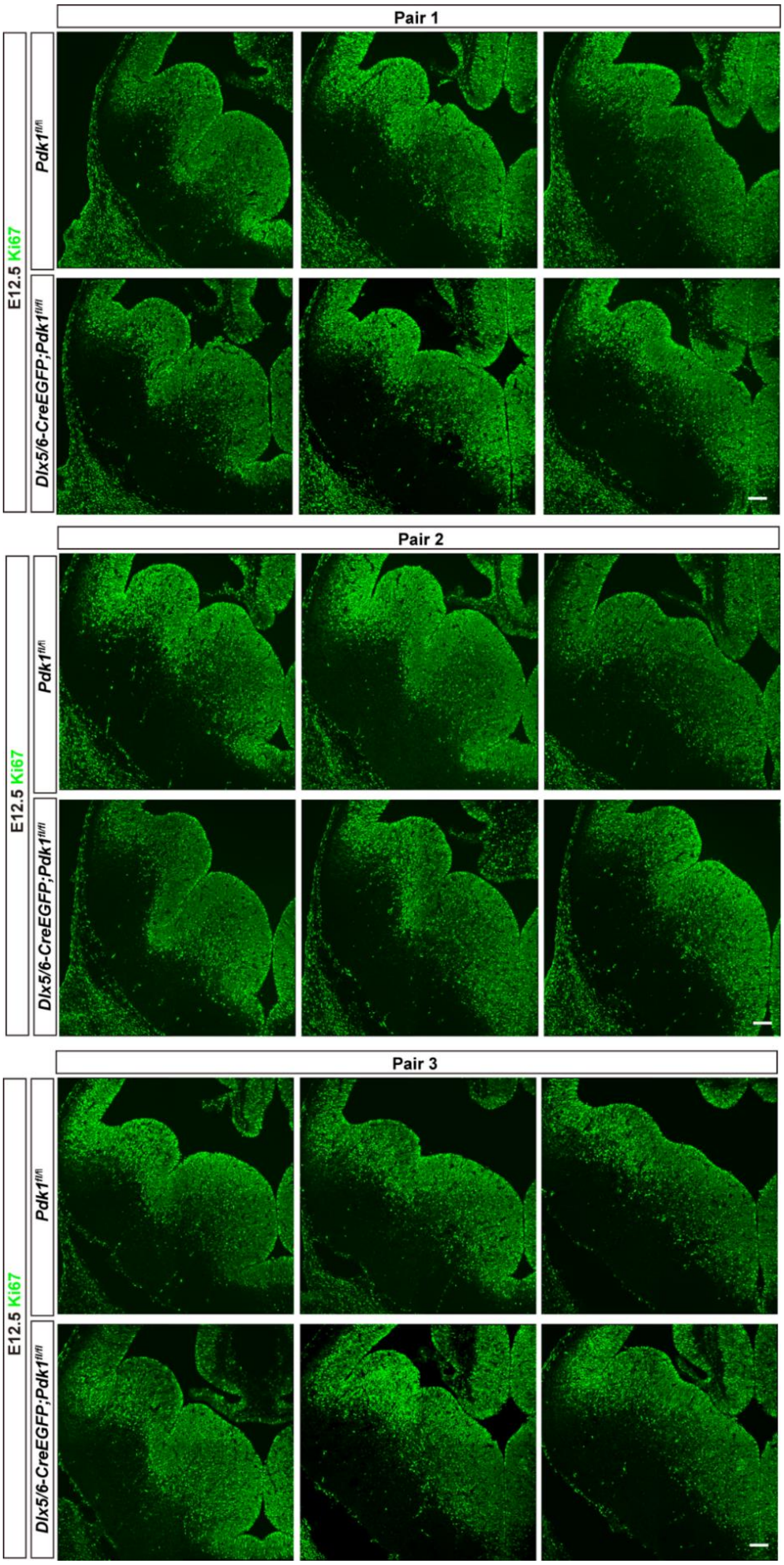

Figure 4O-P Raw Data. Immunofluorescence for Ki67 revealed comparable numbers of Ki67$^+$ cells between *Pdk1* cKO and control mice at E14.5. 4 pair brains from 3 different litters were used for statistical analysis. Quantified data was listed in Raw Data Excel sheet12. P = 0.8033. Scale bar, 100 μm.

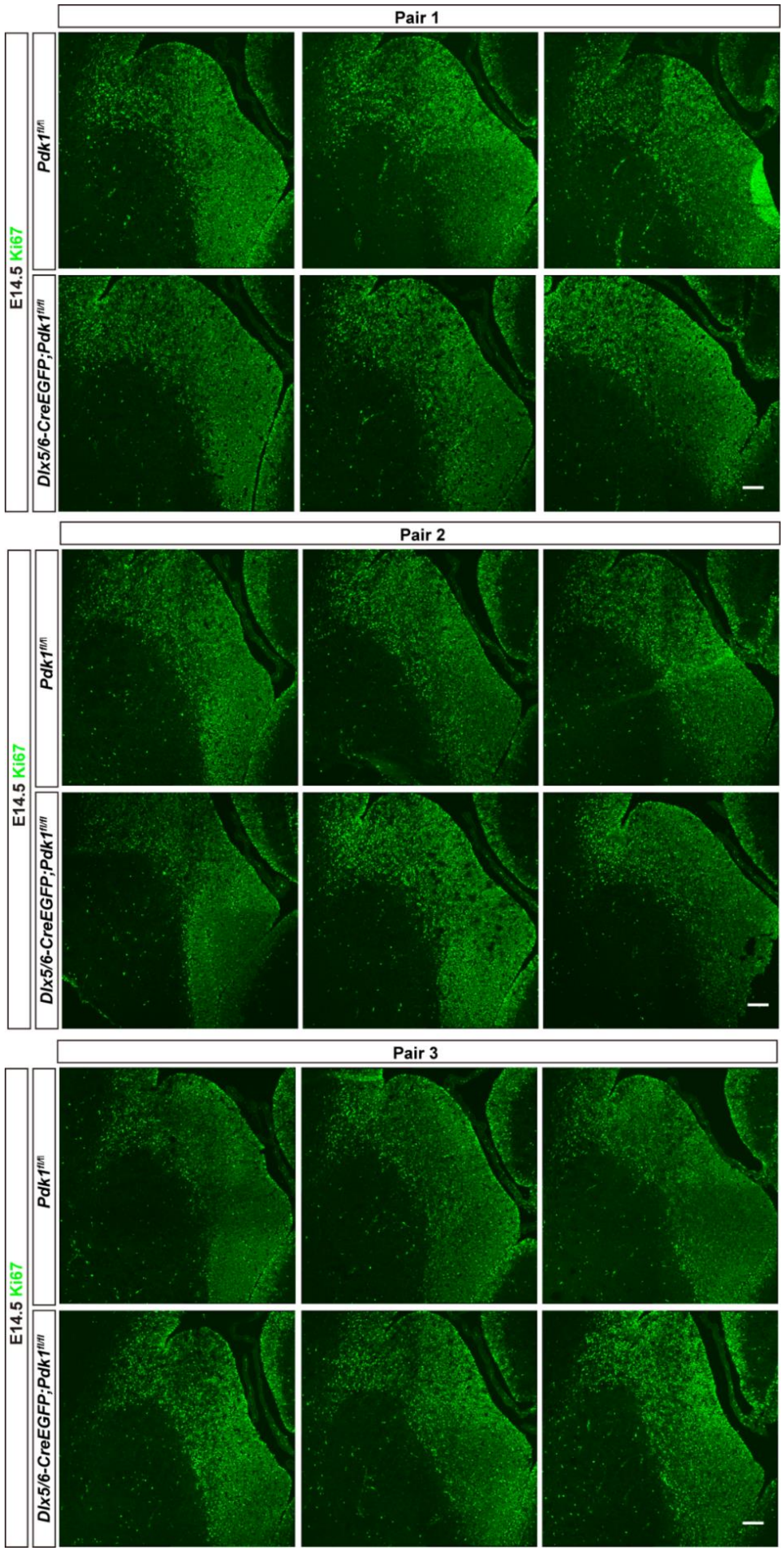

Figure 4Q-R' Raw Data. Immunofluorescence for Ki67 revealed comparable numbers of $Ki67^+$ cells between *Pdk1* cKO and control mice at E16.5. 6 pair brains from 4 different litters were used for statistical analysis. Quantified data was listed in Raw Data Excel sheet12. P = 0.9003. Scale bar, 100 μm.

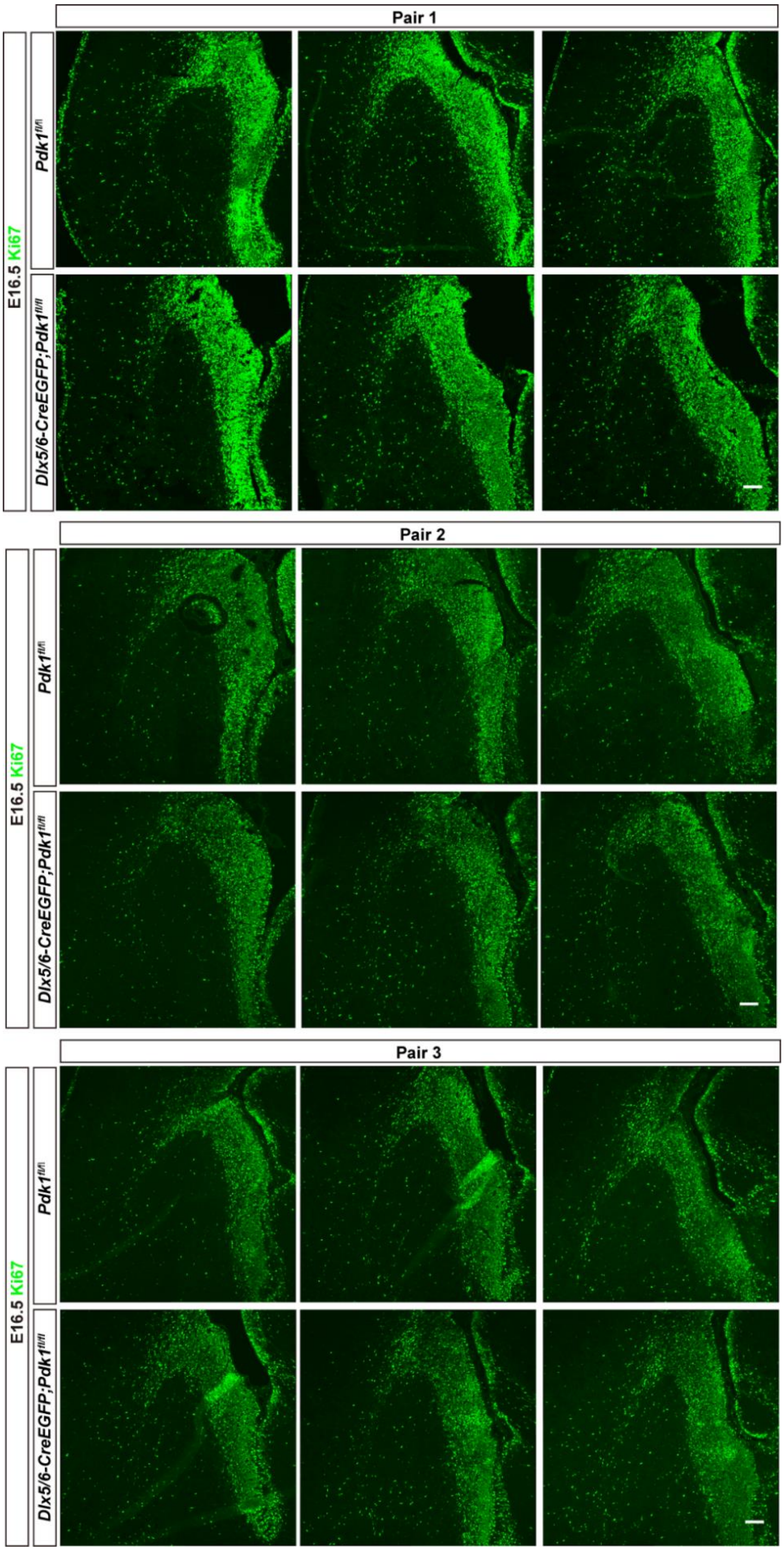

Figure 5A-B' Raw Data. Immunofluorescence showing that the number of Caspase-3^{+} cells was no differences in the subpallium of *Pdk1* cKO and control mice at E12.5. 3 pair brains from 3 different litters were used for statistical analysis. Quantified data was listed in Raw Data Excel sheet13. *P* = 0.9231. Scale bar, 100 µm.

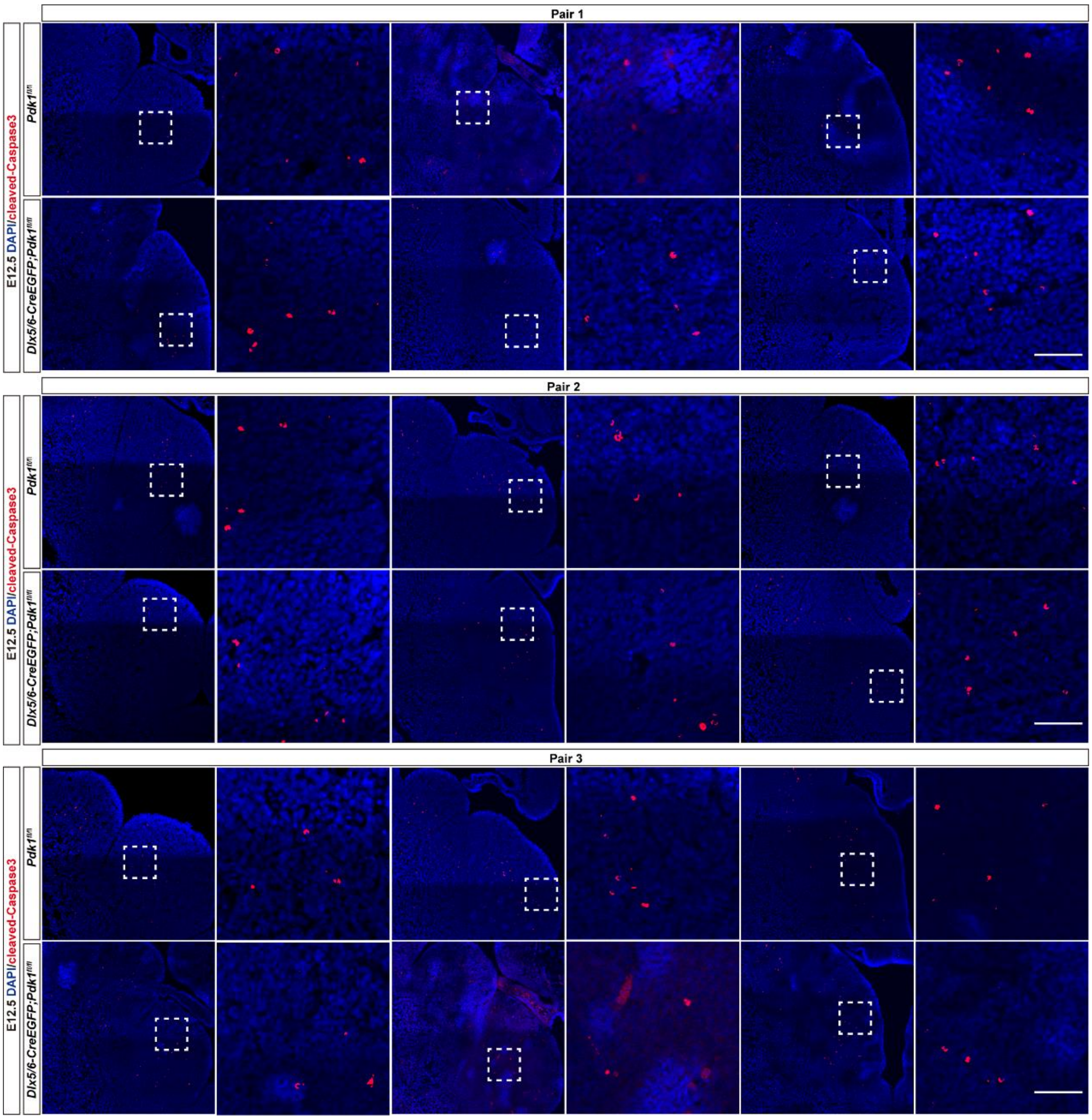

Figure 5C-D' Raw Data. The number of Caspase-3$^+$ cells was significantly increased in the subpallium of *Pdk1* cKO compared with control mice at E14.5. 3 pair brains from 3 different litters were used for statistical analysis. Quantified data was listed in Raw Data Excel sheet13. P = 0.0048. Scale bar, 100 μm.

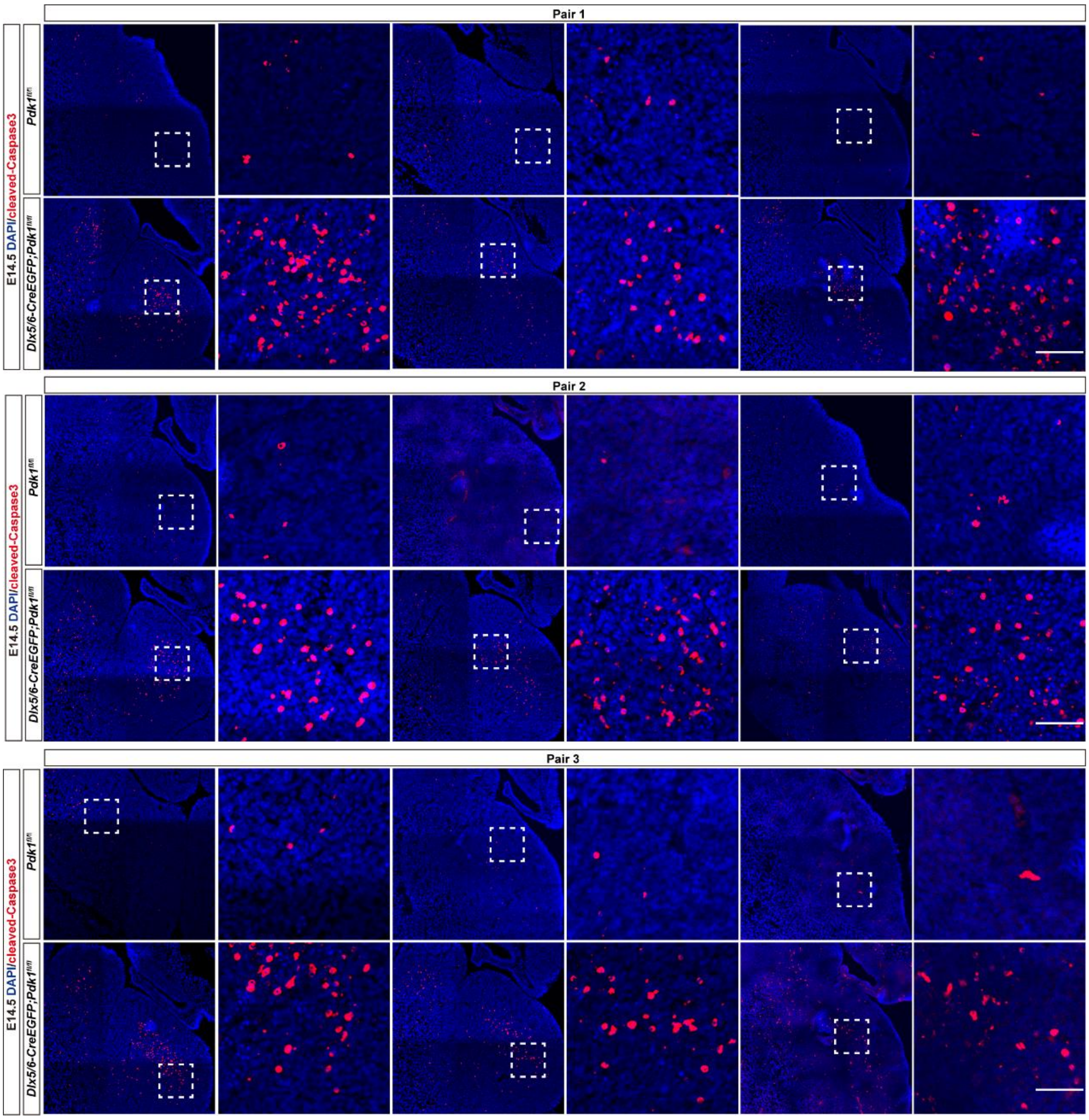

Figure 5E-F' Raw Data. Increased number of Caspase-3⁺ cells in the subpallium of *Pdk1* cKO mice compared to control mice at E16.5. 3 pair brains from 3 different litters were used for statistical analysis. Quantified data was listed in Raw Data Excel sheet13. *P* = 0.0114. Scale bar, 100 µm.

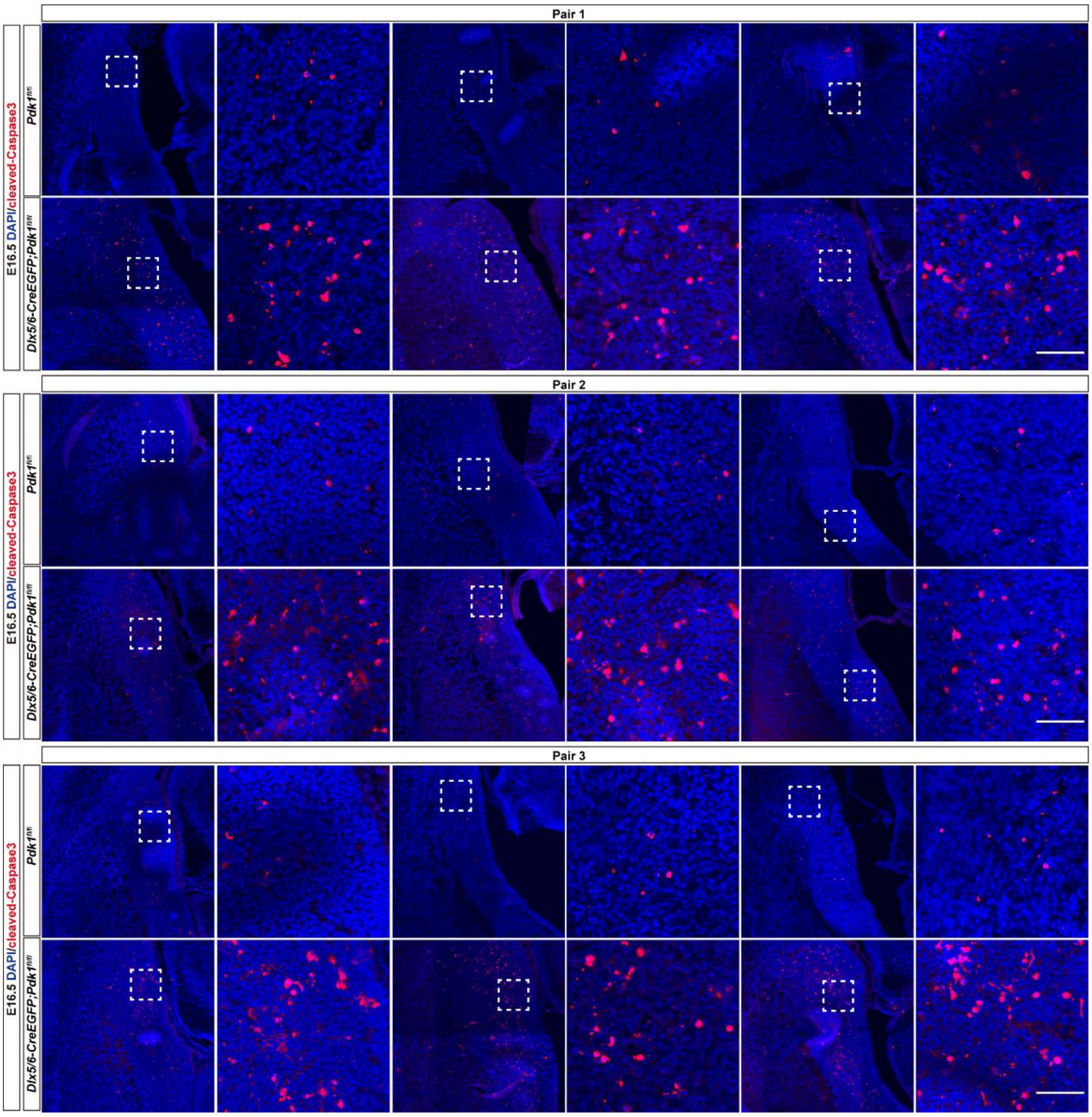

Figure 5G-H' Raw Data. Immunofluorescence for Caspase-3 showed that the number of Caspase-3+ cells was increased in the cortex of *Pdk1* cKO mice compared with control mice at P6. 3 pair brains from 3 different litters were used for statistical analysis. Quantified data was listed in Raw Data Excel sheet13. *P* =0.000037. Scale bar, 100 μm.

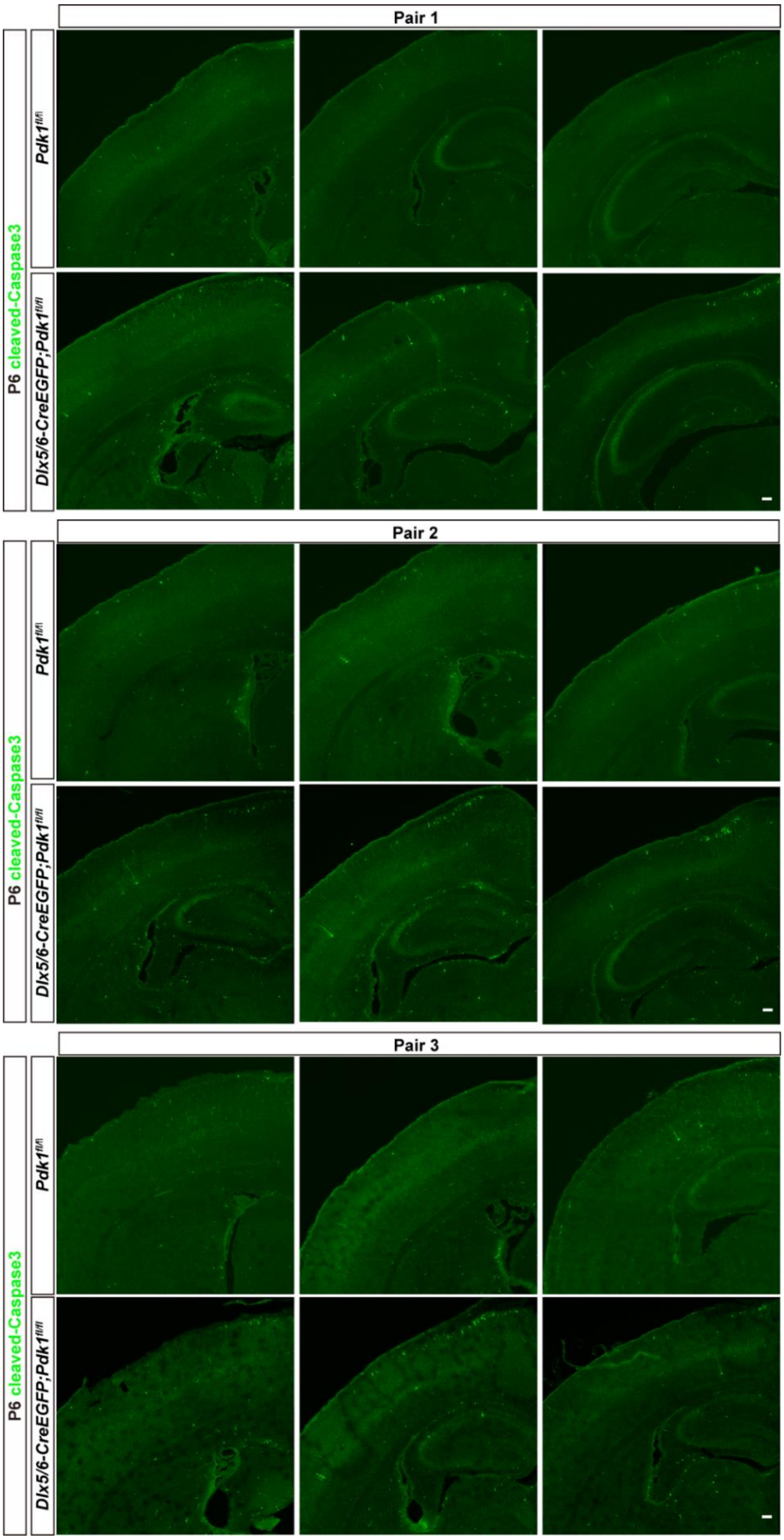

Figure 6A-B Raw Data. There were no differences on the relative expression levels of total AKT in the subpallium between *Pdk1* cKO and control mice at E16.5. 4 pair brains form 3 different litters were analyzed. Quantified data for protein expression levels were listed in Raw Data Excel sheet14. *P* = 0.1186.

Figure 6C-D Raw Data. The relative expression levels of p-AKT^{Thr308} was significantly reduced in the subpallium at E16.5. 4 pair brains form 3 different litters were analyzed. Quantified data for protein expression levels were listed in Raw Data Excel sheet15. *P* = 0.0027.

Figure 6E-F Raw Data. The relative expression levels of p-AKT^{Ser473} was obviously increased in the subpallium of *Pdk1* cKO mice compare to control mice at E16.5. 4 pair brains form 3 different litters were analyzed. Quantified data for protein expression levels were listed in Raw Data Excel sheet16. *P* =0.0153.

Figure 6G-H Raw Data. There were no significant differences in the relative expression levels of total GSK3β in the subpallium between *Pdk1* cKO and control mice at E16.5. 4 pair brains form 3 different litters were analyzed. Quantified data for protein expression levels were listed in Raw Data Excel sheet17. *P* = 0.5900.

Figure 6I-J Raw Data. The p-GSK3β^{Ser9} level was significantly decreased in the subpallium of *Pdk1* cKOs compared to control mice at E16.5. 4 pair brains form 3 different litters were analyzed. Quantified data for protein expression levels were listed in Raw Data Excel sheet18. *P* = 0.0273.

Figure 6K-L Raw Data. There were no significant differences in the relative expression levels of PTEN in the subpallium between *Pdk1* cKO and control mice at E16.5. 4 pair brains form 3 different litters were analyzed. Quantified data for protein expression levels were listed in Raw Data Excel sheet 19. *P* = 0.8366.

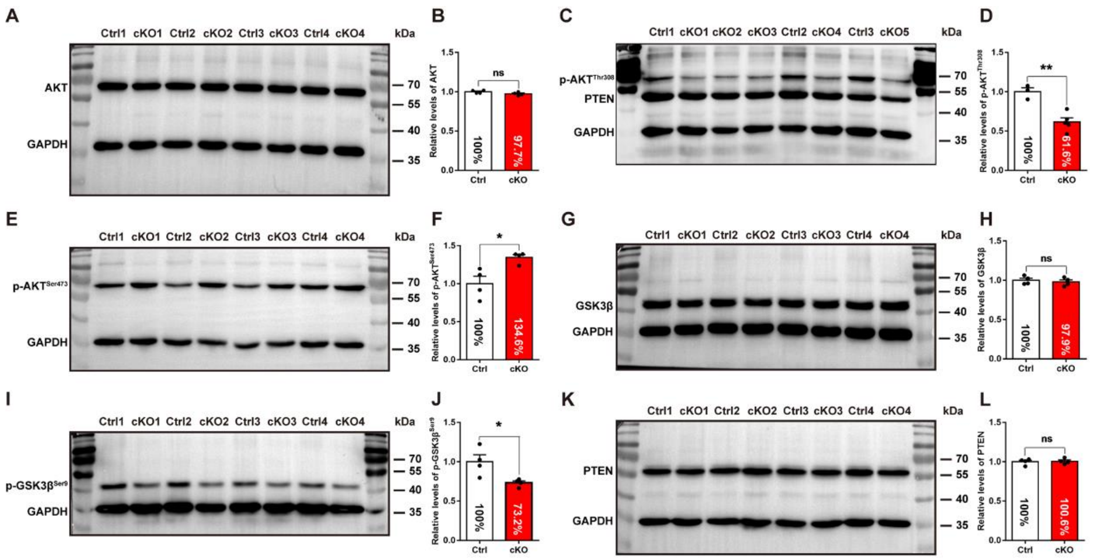

Supplement: Supplementary file 2 — Additional file 2. Raw data supplementary materials [file 13041_2020_604_MOESM2_ESM.pdf]
